# Development of a Flow-free Gradient Generator Using a Self-Adhesive Thiol-acrylate Microfluidic Resin/Hydrogel (TAMR/H) Hybrid System

**DOI:** 10.1021/acsami.1c04771

**Published:** 2021-06-03

**Authors:** Anowar H. Khan, Noah Mulherin Smith, Michael P. Tullier, B. Seth Roberts, Derek Englert, John A. Pojman, Adam T. Melvin

**Affiliations:** † Department of Chemistry, Louisiana State University, Baton Rouge 70803, Louisiana, United States; ‡ Cain Department of Chemical Engineering, Louisiana State University, Baton Rouge 70803, Louisiana, United States; § Chemical and Materials Engineering, University of Kentucky, Paducah 42002, Kentucky, United States

**Keywords:** thiol-ene chemistry, hydrogel, adhesive strength, gradient generator, microfluidics, soft lithography, bacterial chemotaxis

## Abstract

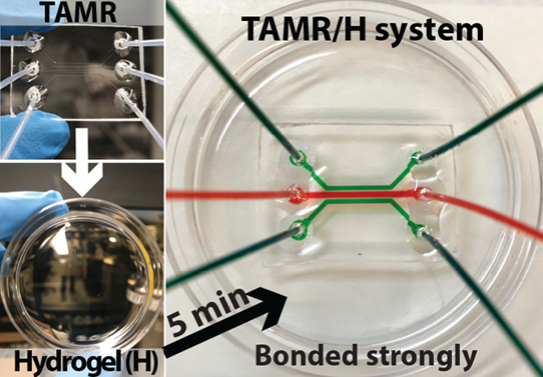

Microfluidic gradient generators have been used to study cellular migration, growth, and drug response in numerous biological systems. One type of device combines a hydrogel and polydimethylsiloxane (PDMS) to generate “flow-free” gradients; however, their requirements for either negative flow or external clamps to maintain fluid-tight seals between the two layers have restricted their utility among broader applications. In this work, a two-layer, flow-free microfluidic gradient generator was developed using thiol-ene chemistry. Both rigid thiol-acrylate microfluidic resin (TAMR) and diffusive thiol-acrylate hydrogel (H) layers were synthesized from commercially available monomers at room temperature and pressure using a base-catalyzed Michael addition. The device consisted of three parallel microfluidic channels negatively imprinted in TAMR layered on top of the thiol-acrylate hydrogel to facilitate orthogonal diffusion of chemicals to the direction of flow. Upon contact, these two layers formed fluid-tight channels without any external pressure due to a strong adhesive interaction between the two layers. The diffusion of molecules through the TAMR/H system was confirmed both experimentally (using fluorescent microscopy) and computationally (using COMSOL). The performance of the TAMR/H system was compared to a conventional PDMS/agarose device with a similar geometry by studying the chemorepulsive response of a motile strain of GFP-expressing *Escherichia coli*. Population-based analysis confirmed a similar migratory response of both wild-type and mutant *E. coli* in both of the microfluidic devices. This confirmed that the TAMR/H hybrid system is a viable alternative to traditional PDMS-based microfluidic gradient generators and can be used for several different applications.

## Introduction

Microfluidic gradient generators have become a mainstay in many research labs due to their ability to precisely control both spatial and temporal dynamics of the chemical and physical microenvironment.^1−3^ Existing microfluidic gradient generating devices can be grouped into two categories: flow-based and flow-free.^4^ Flow-based systems rely on perpendicular diffusion of chemicals directly in the fluidic channel to develop a gradient, whereas flow-free devices develop a gradient via chemical diffusion through a diffusive media such as a hydrogel. First-generation flow-based gradient generators including parallel serpentine channels and in-line channel merging allowed for length-scale diffusion of biomolecules to create chemical gradients perpendicular to the direction of flow.^5−7^ These devices facilitated numerous chemical and biological studies, especially in the field of directed cellular migration to soluble chemical cues or chemotaxis. These flow-based devices were used to study the chemotactic response of bacteria, neutrophils, fibroblasts, and cancer cells.^1,8^ However, with some exceptions reported by Zhang et al.,^9^ one limitation associated with these devices was the need for direct fluid flow over the cells. This resulted in the removal of suspension cells (e.g., bacteria)^10^ or shear effects in adherent cells^11^ (e.g., fibroblasts), which would either complicate the biophysical analysis or potentially alter the biological response of the cells.^11,12^ Recent work to overcome this limitation includes the development of flow-free microfluidic gradient generators. These devices often contain three parallel channels that allow for the orthogonal diffusion of biomolecules from two outer flow channels into a center “flow-free” channel.^10,13,14^ They can be fabricated solely from diffusive hydrogels or utilize a combination of two separate layers (e.g., a hydrogel and a polymer). Microfluidic devices, such as those developed by Cheng et al.^13^ and Berendsen et al.,^14^ directly imprinted the fluidic channels into either agarose or a gelatin hydrogel followed by physical clamping of the device to a glass substrate. This approach has seen great success in recent years with studies in bacterial, neutrophil, and algal chemotaxis. In a recent work, Kong et al.^15^ developed another type of device where a transparent fluidic layer was attached to a glass slide by double-layer adhesive tape and a diffusive agarose hydrogel layer was placed over the fluidic layer. Instead of using physical clamping, a fluid-tight channel was obtained by placing a cover glass layer over the agarose hydrogel, which was attached to the bottom layer by double-layer adhesive tape and polydimethylsiloxane (PDMS) pillars. An alternate device design was utilized by Ahmed et al.,^16^ Salek et al.,^17^ and Rahman et al.^18^ where fluidic channels were imprinted into PDMS, which was then coupled with an agarose hydrogel to study bacterial chemotaxis. The hydrophobic nature of PDMS^19^ and the hydrophilic nature of hydrogels^20^ result in no true adhesive interactions between the two layers or with a glass substrate. Therefore, this approach requires negative flow, external clamps, adhesive tape, surface treatment with oxygen plasma, or surface functionalization with different molecules to maintain fluid-tight seals between the hydrogel and glass or the PDMS and the hydrogel.^13−16,18,21^ Proper assembly of these devices requires great operational accuracy, as the fluidic channels can collapse, the hydrogel can rupture, or pressure differences across the device can bias cellular behavior.^14,21^ Similarly, fluid leakage can occur if insufficient pressure is applied during device assembly.^21^ These challenges with device assembly and operation often require substantial expertise in device construction and operation which can limit their overall utility in other labs.

While PDMS is the most commonly used polymer to fabricate microfluidic devices, recent work has identified alternative polymers capable of making fluidic channels. As with PDMS, these polymeric alternatives are cheap, are readily available, are capable of rapid polymerization and molding, possess good optical clarity, are compatible with undergoing surface modification, and are biocompatible.^22,23^ Materials such as poly(methyl methacrylate), polycarbonate, and cyclic olefins copolymers (COC/COP) have been utilized in developing microfluidic devices; however, each of these materials has limitations in the abovementioned criteria.^22−25^ Recent work by Bounds et al. and Tullier et al. aimed to overcome some of these challenges by developing a novel polymeric material to replace PDMS using thiol-ene chemistry,^23,25,26^ which allows for polymerization by applying UV-light (photopolymerization)^27,28^ or by a base-catalyzed Michael addition.^23,26,29,30^ Both of these techniques resulted in highly cross-linked thermoset polymers in short time scales (∼30 min) with close to 100% monomer conversion.^23,31,32^ One limitation of photopolymerized materials is the generation of exogenous reactive radicals and reactive macromers during the polymerization process and some of these reactive radicals or the accumulation of unconsumed initiators left in the system that can adversely affect cell viability for biological applications.^33^ Conversely, a base-catalyzed Michael addition is a mild (pH 7.4–8) reaction occurring between a thiol and electron-deficient enes (e.g., acrylates) containing an electron-withdrawing carbonyl group, which eliminates the possibility of the residual unreactive initiator or macromers in the system since Michael addition reaction takes place simply via nucleophilic addition between thiolate to acrylate groups, leaving no reactive species.^23,29,30,34,35^ An advantage with polymers using a base-catalyzed Michael addition is that the chemistry can be easily tuned to yield a rigid fluidic channel or cell-compatible hydrogels. One area that has yet to be explored is the combination of these two polymeric materials to produce a microfluidic gradient generator.

The goal of this study is to develop a self-adhesive thiol-acrylate microfluidic resin/hydrogel (TAMR/H) hybrid system capable of producing flow-free chemical gradients. Of the many advantages of the TAMR/H system, the most prominent one is that the two separate layers are adhesive with each other, which eliminates the need for external clamping or negative flow, significantly reducing complications associated with device operation. Moreover, the TAMR/H is the only microfluidic gradient generator where the fluidic layer binds with the hydrogel within 5 min, eliminating the need of expensive instruments such as a plasma cleaner or complex monomer modification. All the components of the TAMR/H system are commercially available and can be used without any additional modification or purification. Unlike PDMS, TAMR is hydrophilic and both the TAMR and hydrogel surface can be modified relatively easily compared to PDMS.^23,25^ This is because both the TAMR and hydrogel are made with thiol and acrylate monomers which can be functionalized with different groups in time of need. The microfluidic or rigid PDMS-like TAMR contains negatively imprinted channels.^23,25^ The diffusive layer (or porous hydrogel, H) was synthesized to facilitate the passive diffusion of biomolecules. The TAMR/H system was compared to a control, flow-free microfluidic device consisting of a fluidic channel imprinted into PDMS coupled with an agarose slab encased in a Plexiglas chamber. As a proof of concept, the TAMR/H was used to study the chemorepulsive response (e.g., migrating down the gradient away from the chemical source) of a motile strain of green fluorescent protein (GFP)-expressing *Escherichia coli* (*E. coli*). Bacterial chemotaxis has been extensively studied in both flow and flow-free devices due to their high motility and rapid response to external stimuli, resulting in either a chemotactic or chemorepulsive response.^4,36−39^

## Materials and Methods

### Chemicals

Polyethylene glycol diacrylate (PEGDA, Mn 700), trimethylolpropane ethoxylate triacrylate (TMPETA, Mn 912), diethylamine (DEA), 5,6-carboxyfluorescein (Mw 376), and nickel sulfate heptahydrate (NiSO_4_·7H_2_O) were purchased from Sigma-Aldrich. Ethoxylated trimethylolpropane tri(3-mercaptopropionate) (ETTMP, Mn 1300) was generously donated by Evans Chemetics LP. Pentaerythritol tri-tetraacrylate (tetra- to tri- acrylate ratio ∼1 to 1) (PETIA, Mn 325) was purchased from Allnex. Trimethylolpropane tris(3-mercaptopropionate) (TMPTMP, Mw 398.5) was purchased from TCI (Figure S1). Biotechnology-grade phosphate-buffered saline (PBS) tablets were purchased from VWR Life Science. Erythromycin, LB broth, and dl-lactate were bought from Alfa Aesar. Dextrose was bought from VWR analytical DBH. Tryptone was purchased from BD Bioscience, and l-methionine was purchased from Acros Organic. Biograde sodium chloride (NaCl) was purchased from Fisher Scientific.

### Cell Culture and Reagents

Two strains of GFP-expressing *E. coli* RP437 (wild type) for chemotaxis and the knock out mutant that cannot sense or respond to Ni^2+^ and aspartate^36,40^ were maintained in agar plates. Both strains required frequent cell culturing at least once every 14 days, but often at a higher frequency of about 10 days to maintain optimal motility and fluorescence. Prior to plating, cells were grown in LB broth (20 g of LB broth and 10 g of dextrose dissolved in 1 L of DI water and later filtered with a 500 mL bottle top filter, 0.2 μm under biosafety hood) for 10–14 h at 32 °C and 130 rpm. Afterward, *E. coli* were plated on agar and cultured at 32 °C for 12 h before storage at 4 °C for future use. Prior to the migration experiments, bacterial cells were grown in LB broth under similar conditions as before. Subsequently, 1 mL of overnight culture was inoculated into 20 mL of tryptone broth (TB; 10 g of tryptone and 8 g of NaCl were dissolved in 1 L of DI water and later filtered with a 500 mL bottle top filter, 0.2 μm under biosafety hood) and allowed cells to grow approximately for 4 h at 32 °C and 130 rpm before using cells for migration experiments. During the culturing period, maintaining a temperature of 32 °C is important as deviation from this temperature would decrease motility or negatively affect fluorescence. All buffers and plates were supplemented with erythromycin at 1.5–3 μg/mL to maintain the fluorescence of the cells.

### Synthesis of TAMR

The TAMR used in this study is similar in nature to the material developed by Bounds et al.^23^ Our current TAMR/H hybrid system used a TAMR layer that was synthesized with the 50% excess acrylate group using thiol-ene chemistry.^26^ Details of the synthesis method for TAMR can be found in the Supporting Information in addition to the structures of the monomers used in the synthesis (Figure S1).

### Synthesis of the Hydrogel (H) for the TAMR/H System

A 15% (w/w) hydrogel was synthesized for the TAMR/H system following a Michael addition reaction (Scheme S1).^30,35^ Aqueous NaOH (20 μL, 5 M) was added to 5 g of 1× PBS (each PBS tablet was dissolved in 100 mL of DI water to obtain a buffer solution which contains 137 mM sodium chloride, 2.7 mM potassium chloride, and 10 mM phosphate buffer) which made the reaction mixture basic to initiate the Michael addition reaction. An appropriate amount of the acrylates, PEGDA and TMPETA (in 50:50 ratio by the number of functional groups), were then added to the 1× PBS and subsequently vortexed for 5 s to disperse the acrylate monomers homogeneously in the buffer solution. Finally, ETTMP was added to the solution, and the complete reaction solution was then vortexed vigorously for 30 s before pouring into a 60 mm Petri dish and left undisturbed until reaction reached completion. The gelation time was measured by the tube inversion method as previously described.^30,41^ This time was identified as the time at which a bubble would no longer rise in the hydrogel even if hydrogel was inverted.^30,41^

### TAMR/H Microfluidic Device Design and Fabrication

**Figure 1:**
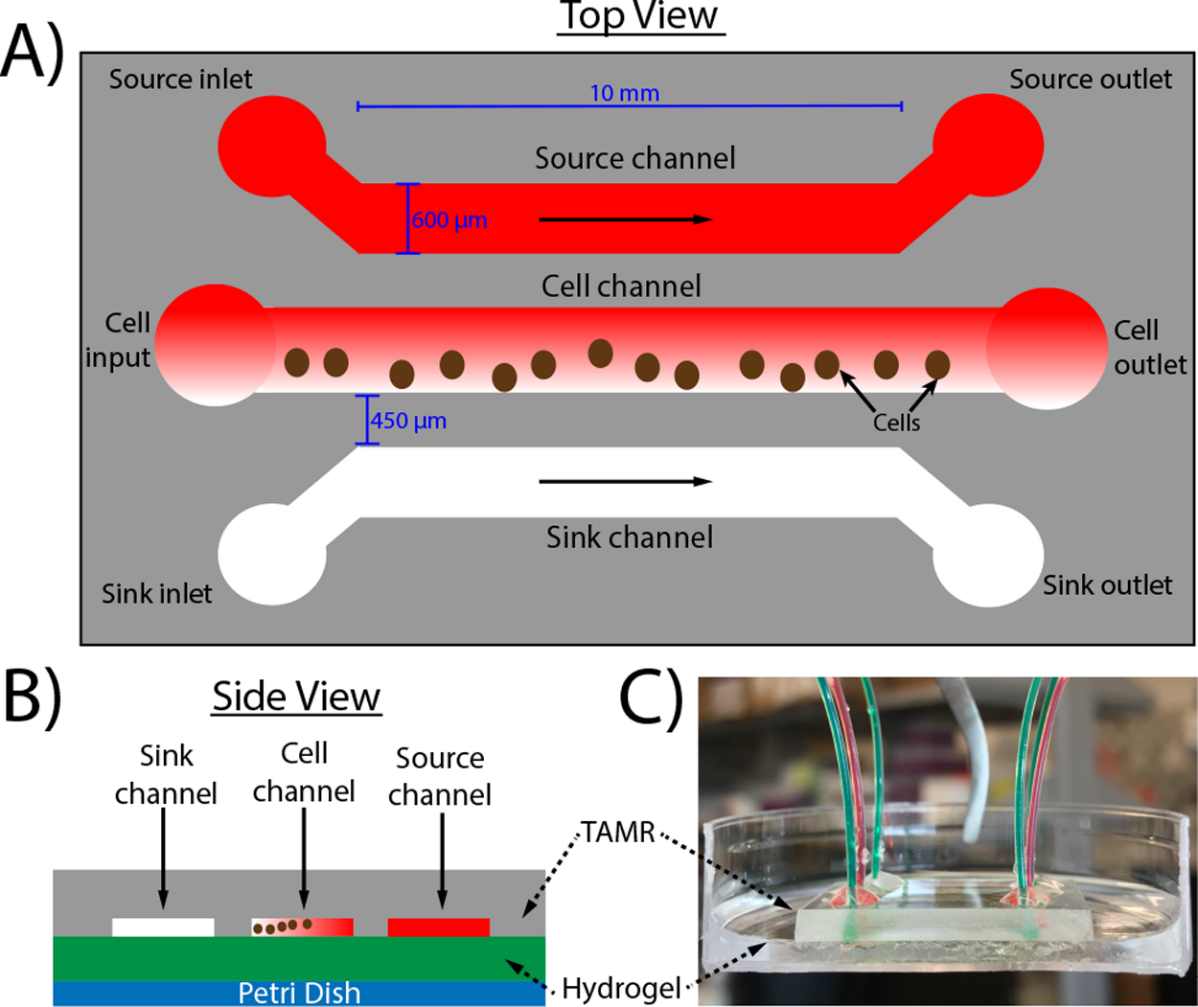
TAMR/H microfluidic device. (A) Schematic of the device geometry imprinted into the top TAMR layer. (B) Side-view schematic of the assembled TAMR/H device consisting of the top TAMR layer bound to the bottom hydrogel (H) layer. (C) Cross section of an actual TAMR/H device.

The TAMR/H microfluidic device (Figure 1) consisted of two separate layers: a bottom layer thiol-acrylate hydrogel to facilitate chemical diffusion and a top TAMR layer into which three parallel channels were imprinted (Figure 1A). The TAMR layer consisted of three 600 μm-wide parallel channels with a total operating length of 10 mm and a channel height of 150 μm (Figure 1A). The outermost channels were designed to accommodate flow to perfuse the system with a source and sink to generate a gradient in the center flow-free channel. The outermost channels were spaced 450 μm from the center channel. Details regarding the fabrication of the master silicon wafer can be found in the Supporting Information. Individual TAMR devices were cut out with an X-Acto knife, followed by drilling the inlet and outlet ports using a DeWalt 20v Max equipped with a 3/64 in. Dremel drill bait. Tygon tubing (0.022″ inner diameter × 0.042″ outside diameter of tubing, Cole-Parmer Instrument Company) was inserted into the six inlet and outlet ports and sealed in place by pouring ∼500 μL of liquid TAMR around the port, which solidified in ∼5 min. The TAMR layer, with tubing in place, was placed on the hydrogel surface and allowed to interact with the hydrogel for 5 min before using it as a fluid-tight microfluidic system (Figure 1C). For long-term experiments, the hydrogel layer of the TAMR/H system that was not covered by TAMR was covered with deionized (DI) water to prevent evaporation of water from the hydrogel matrix. Fabrication and assembly of the control microfluidic device (PDMS/agarose, Figure S2) are described in the Supporting Information.

### Mechanical Stability Testing of the Hydrogel

The mechanical stability of the hydrogel (H) was evaluated using both degradation and rheological characterization. Degradation of the hydrogel was carried out gravimetrically in DI water (pH 8.15) or chemotactic buffer (CB, pH 7.41).^30^ The hydrogel was synthesized in 8 mm glass vials, and the initial weight (*W*_0_) was recorded. Once the hydrogel was solidified, an equal amount of DI water or CB was incorporated and incubated at 37 °C and 5% CO_2_ in the humidified incubator. Every 24 h, the solution (DI water or CB) was carefully removed from the hydrogel surface, and the weight of the gel was taken as *W*_f_. Degradation was monitored at different time intervals using the below equation as previously reported.^30,42^
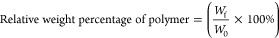
1

To determine the rheological properties of the hydrogel, the storage modulus (*G*′) and loss modulus (*G*″) of the hydrogel were determined in a frequency sweep range of 0.682–62.8 radians per second and a constant shear strain amplitude of 2%. A small amplitude oscillatory shear was applied to a piece of hydrogel sample (8 mm) using an 8 mm parallel disk geometry at 25 °C. The complex modulus (G*) and tan δ values were estimated using storage (*G*′) and loss modulus (*G*″) values obtained from rheological measurement as previously described.^30,43^
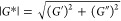
2

### Adhesion Strength Testing between the TAMR and Hydrogel

To determine the adhesion strength between the TAMR and hydrogel, a 3.5 mm-thick slab of hydrogel was synthesized on a sheet of Plexiglas and a piece of TAMR (22 mm × 32 mm) was glued on another sheet of Plexiglas using 5 min epoxy glue. Both layers were brought into contact and left for 5 min before measuring the adhesion strength using a single-lap shear test on an Instron equipped with a 2 kN load cell at a constant ramping rate of 2 mm/min for the 22 mm × 32 mm adhesive area.^44,45^ Adhesion strength was also evaluated using a maximum flow test. In this test, both experimental (TAMR/H) and control (PDMS/agarose) devices were assembled and continuously infused with DI water spiked with red food dye in the middle channel at different flow rates for 5 min using a syringe pump. The microfluidic channels were monitored using a Zeiss Primo Vert inverted phase contrast microscope at a 4× objective to spot any fluid leakage.

### Diffusion Coefficient Approximation for the Hydrogel Used in the TAMR/H System

In order to estimate the rate of mass transfer, the diffusion coefficient of bromothymol blue (MW: 624 g/mol) in the hydrogel was measured by imaging the concentration profile as a function of time as previously described.^30,46,47^ A 15% w/w hydrogel was synthesized in a plastic UV cuvette cell and allowed to solidify. Then, 200 μL of 1% w/w aqueous bromothymol blue solution was carefully placed on top of the hydrogel. Images were collected every 5 min for 500 min using a Nikon D3200 (072 DiII TAMRON 18-270 mm). ImageJ (NIH) was used to analyze the gray level intensity versus vertical position in the cuvette as previously described.^30,47,48^ The analysis assumed that the gray level intensity was proportional to dye concentration. The gray level profile along the vertical axis was fit to the following equation, where *y* is the position, *t* is time, and *D* is the diffusion coefficient
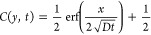
3

At each time point, the gray level intensity profile was fitted for  using KaleidaGraph 4.5 (Reading, PA). The values of 4*Dt* were then plotted against time to generate a line where the 0.25 × slope provided the value of the diffusion coefficient.

### Experimental and Computational Validation of Mass Transfer in the Microfluidic Devices

Mass transfer in the TAMR/H device was experimentally visualized via the diffusion of 5,6-carboxyfluorescein (FAM, MW: 376.5 Da). A 10 μM FAM solution in CB (1% PBS mixed with 0.1 mM EDTA, 0.01 mM l-methionine, and 10 mM dl-lactate) was infused into the source channel of the TAMR/H device at a flow rate of 15 μL/min. Simultaneously, a “blank” CB solution was infused into the sink channel at the same flow rate. The center channel was filled with CB and then closed off with blunted 23-gauge needles. The diffusion of FAM was visualized using a Leica DMi8 inverted fluorescence microscope outfitted with an FITC filter cube using the 5× objective. Brightfield (BF) and fluorescence (FITC) images were acquired at 45 and 20 ms exposure times, respectively, every 2 min for 12 h. The FAM gradient was quantified using LASX software performing a line scan across the width of the channel at defined time intervals to identify fluorescent intensity as a function of distance. A similar experiment was performed using the control PDMS/agarose device.

COMSOL Multiphysics was used to construct a 3D device geometry similar to the TAMR/H system. The initial simulation was built for the PDMS/agarose device, which has defined parameters for both PDMS and agarose. The diffusion coefficient (*D*) used for the COMSOL simulations for the PDMS/agarose device was the value for nickel (Ni^2+^) cations in an agarose hydrogel, which is 6.54 × 10^–6^ cm^2^/s. This value of *D* is similar in magnitude for other small molecules (e.g., FAM) in agarose hydrogels and in water.^13,16,49,50^ Line scans were used to compare the gradient shape, and literature values were used to provide an estimate of magnitude. For all simulations, water was used as the transport medium in the channels. The diffusion coefficient and porosity of the TAMR/H hydrogel within the COMSOL simulations were tuned to match the trend established by the experimental line scans. Simulations were performed under identical conditions to those used in the fluorescent gradient characterization. The TAMR/H device yielded a diffusion coefficient for Ni^2+^ and porosity of 9.5 × 10^–7^ cm^2^/s and 0.2, respectively, as determined via the COMSOL Multiphysics model.

### Chemotaxis Experimentation and Image Analysis

Prior to experimentation, *E. coli* was grown in LB broth for 10–14 h (overnight) in a shaker at 32 °C and 130 rpm. To deprive these cells of nutrients and increase motility, 1 mL of the overnight culture was inoculated into 20 mL of blank TB broth and allowed it to grow approximately for 4 h. The optical density (OD) of the culture was measured using a UV/vis spectrophotometer (Beckman Coulter DU 730) at 600 nm. The culture (10 mL) with an OD_600_ of 0.4–0.6 was centrifuged at 250*g* for 10 min, and the supernatant was carefully decanted. The cells were resuspended in 10 mL of neutral CB, resulting in a final OD_600_ of 0.5.

Prior to injection of the cells into the device, both devices (TAMR/H and PDMS/agarose) were primed by infusing the source channel with 2 mM Ni^2+^ solution made in CB and the sink channel with only CB at a rate of 15 μL/min to establish a stable chemical gradient of Ni^2+^ across the center flow-free channel. Due to the difference in the diffusion rate of Ni^2+^ in the TAMR/H hydrogel versus the agarose hydrogel, this priming period was ∼4 h for the TAMR/H device and ∼2 h for the PDMS/agarose device. In the case of the no gradient control (random migration), only CB was infused in both channels (source and sink). After the device was primed, the *E. coli* suspension was manually injected into the center channel using a 1 mL syringe followed by capping out the inlet and outlet of the center “flow-free” channel with blunted 23-gauge needles. *E. coli* distribution in the flow-free channel was visualized using a Leica DMi8 inverted fluorescence microscope equipped with an FITC filter cube at 10× objective. BF and fluorescence (FITC) images were acquired at 50 and 150 ms exposure times, respectively, across the entire 10 mm length of the channel. To observe the effect of the 2 mM Ni^2+^ on *E. coli* migration, the cells were exposed to the gradient, and within 20 min of exposure to Ni^2+^ gradient, cells were observed to demonstrate a chemorepulsive response toward the Ni^2+^ gradient. Once the bacterial chemorepulsive response was established, images across the length of the channel were collected.

All fluorescence microscopy images were processed using ImageJ (NIH, USA) to assess cellular distribution in the center channel. Each fluorescence image was converted into a grayscale image, and a threshold was set so that a binary image would be produced to remove any background signal. The ImageJ particle analysis tool was used to identify and quantify the cell position and count. Each image was processed individually, first identifying cells and then identifying the bounds of the center flow-free channel. The center channel was divided into four equal horizontal quadrants (150 μm wide) with quadrant 1 being the closest to the chemical source and quadrant 4 being the furthest from the source and closest to the sink. To validate *E. coli* chemorepulsion, the total number of cells in each quadrant was recorded followed by calculating the percent distribution of the population in each of these quadrants.

### Statistical Analysis of Chemotaxis Data

Data presented in this study were representative of at least three independent experiments, and all values were represented as arithmetic mean ± standard deviation. The statistical difference between different groups was determined by the standard *t*-test using Microsoft Excel where *p*-value < 0.05 was considered as statistically significant (*) and *p* < 0.01 was considered statistically very significant (**), while *p* > 0.05 was considered statistically nonsignificant (ns).

## Results and Discussion

### Synthesis and Characterization of the Hydrogel (H) Component of the TAMR/H Device

The TAMR material in this study was similar to the material developed by Bounds et al.,^23^ so it was not further characterized. The hydrogel (H) was synthesized by cross-linking a trithiol (ETTMP) with diacrylate (PEGDA) and triacrylate (TMPETA), and the reaction was initiated by a small amount of base (Scheme S1).^30,35^ A water-soluble base (NaOH) was used as the reaction occurred in aqueous medium. The hydrogel contained a 15% (w/w) thiol-acrylate polymer with the acrylate groups derived from PEGDA (diacrylate) and TMPETA (triacrylate) in a ratio of 50:50 (by functional groups). The trifunctional acrylate (TMPETA) was introduced into the system to increase the cross-linking density of the hydrogel and provide sufficient stiffness so that the gel would not rupture when combined with the TAMR. The gelation time was determined to be 8.0 ± 2.0 min as determined from 10 replicates. FTIR characterization was performed to validate that the hydrogel was formed by reacting the thiol and acrylate monomers (Figure S3). FTIR data show IR bands that are responsible for the thiol and acrylate groups disappearing from the hydrogel (H) spectrum after gelation. These bands are located at 2560, 1635, 1408, 990, and 810 cm^–1^ which can be indexed to S–H stretching (thiol), C=C stretching (acrylate), =CH_2_ bending, =CH_2_ wagging, and =CH_2_ twisting, respectively similar to results previously described by Khan and colleagues.^30^ These findings confirm that the thiol groups (−SH) of ETTMP were deprotonated into a thiolate (−S^–^) by the base, and this deprotonation allowed for the addition of thiolate groups to the double bonds of the acrylate groups (present in PEGDA and TMPETA) forming a thioether bond. Advantageously, both the TAMR and hydrogel can be synthesized at room temperature (25 °C) and ambient pressure without any further treatment, which made device replication easy and reproducible.

### Degradation and Rheological Analysis to Confirm the Mechanical Stability of the Hydrogel Component of the TAMR/H System

**Figure 2:**
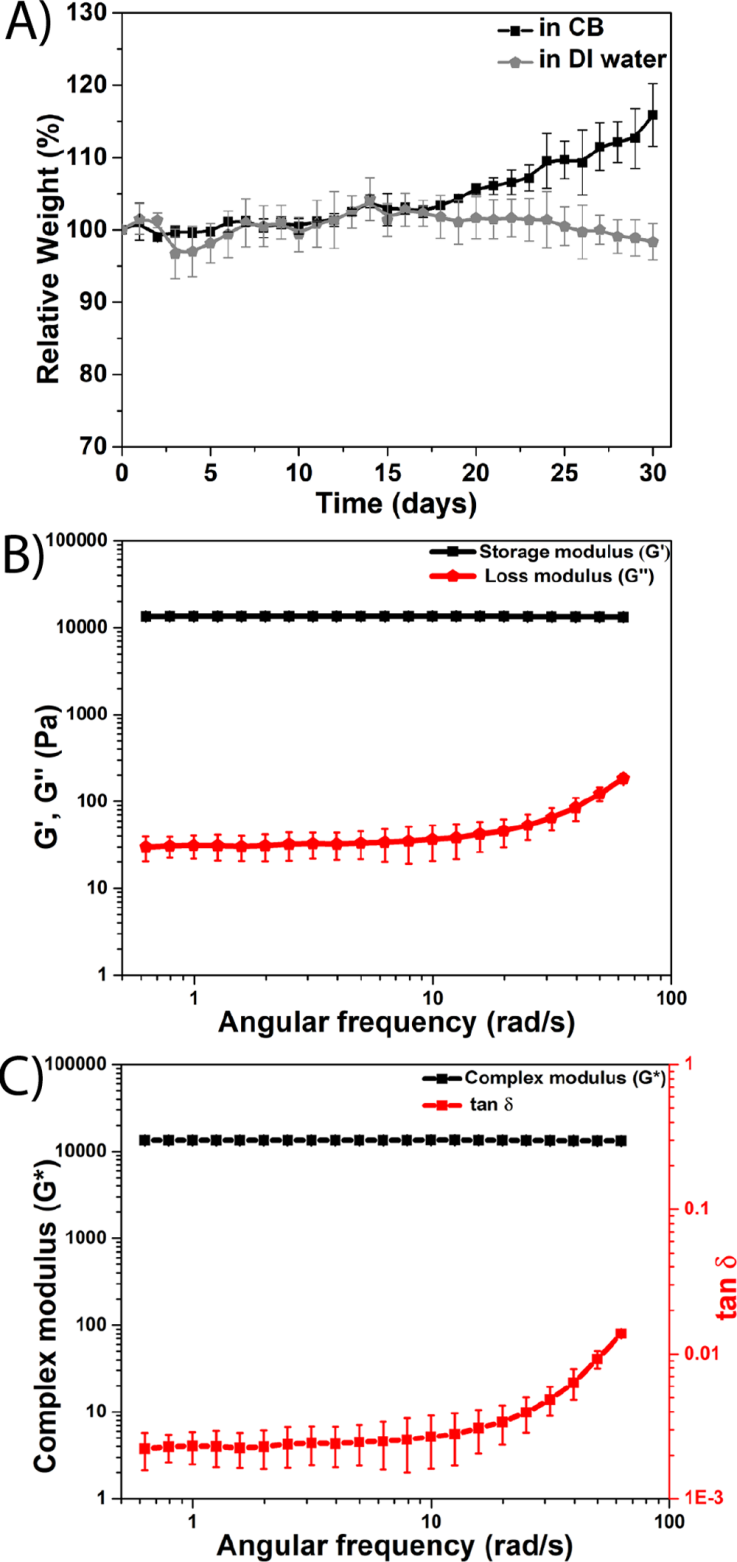
Characterization of mechanical stability of the hydrogel (H) of the TAMR/H system. (A) Degradation profile of 15% w/w covalently cross-linked thiol-acrylate hydrogels in two buffers: CB (pH = 7.41) and DI water (pH = 8.15) at 37 °C. Bulk rheology of the covalently cross-linked thiol-acrylate hydrogel was taken during frequency sweeps (0.682 to 62.8 radians/second) at 25 °C. (B) Comparison between the storage modulus (*G*′, elastic component) and the loss modulus (*G*″, viscous component) during the frequency sweep. (C) Comparison between the complex shear modulus G* (provides insights into hydrogel stiffness) and tan δ (tan δ ∼ 0: purely elastic material and tan δ ∼ 1: viscous liquid) during the frequency sweep.

The stability of the hydrogel component of the TAMR/H device was evaluated using a twofold approach. First, the stability of the hydrogel was assessed using both DI water (pH ∼ 8.15) and CB (pH ∼ 7.41) using a series of degradation studies. The weight loss of the hydrogel in both solvents was monitored for 30 days showing that the hydrogel remained stable for at least 18 days in both DI water and CB as determined by minimal or no weight loss (<5 wt %) (Figures 2A, S4). The hydrogel was found to slowly degrade with <10 wt % loss in DI water and swelling of <15 wt % in CB after 30 days. The observed degradation in DI water and swelling in CB after 18 days can be attributed to the lower pH of the CB in comparison to DI water pH because the rate of ester hydrolysis increases at higher pH (basic media).^30,51^ It was suspected that the CB resulted in slow hydrolysis of the ester bonds of the hydrogel compared to DI water due to the higher pH of the DI water. The high stability of the hydrogel can be attributed to the pH of the degrading solvent and high cross-linking density of the polymer present in the hydrogel based on the hydrolytic degradation of the hydrogel being governed by ester hydrolysis and cross-linking density.^30,52,53^ The cross-linking density of a hydrogel is directly proportional to the polymer content and functionality of the monomers used in the system.^30,41,54,55^ The hydrogel contains a 15% w/w polymer and both a trifunctional acrylate (TMPETA, containing three active arms) and a difunctional acrylate (PEGDA, containing two active arms) as cross-linkers. This resulted in increased cross-linking density, which delayed the degradation of the hydrogel since degradation is inversely proportional to the cross-linking density.^30,53^

This was further verified by evaluating the relationship between the storage modulus *G*′ and degradation time of the hydrogel used in the TAMR/H system and comparing these findings to a thiol-acrylate hydrogel previously reported by Khan et al.^30^ Rheological data showed that the *G*′ of the 15% w/w hydrogel used in the TAMR/H system was ∼13 kPa (Figure 2B), while a previously reported 9.5% w/w hydrogel yielded a value of 0.9 kPa.^30^ The large *G*′ value observed for the TAMR/H system confirms a large cross-linking density compared to similar hydrogels with lower polymer content since cross-linking density is linearly dependent on the storage modulus *G*′.^30,41,56^ Due to a lower cross-linking density, the 9.5% w/w hydrogel degraded completely within 6 days in similar pH media, which is not the case for the TAMR hydrogel as it contains a greater cross-linking density. The rheological data were also used to measure the mechanical stability of the hydrogel against applied shear. The storage modulus *G*′ (elastic component) and loss modulus *G*″ (viscous component) were found to be independent in a lower angular frequency range (0.1–30 rad/s) (Figure 2B), confirming that the hydrogel is fully cross-linked since *G*′ and *G*″ will remain independent over a large frequency change for a fully cross-linked system.^41^ The hydrogel was found to remain highly elastic and intact over large frequency changes of shearing as evidenced by the *G*′ value remaining 2–3 orders of magnitude greater than the *G*″ value (Figure 2B) and the tan δ value remaining close to zero (Figure 2C). An elastic material *G*′ will remain significantly larger than the *G*″, and tan δ will remain close to zero over a large frequency change.^41,43^ The hydrogel storage modulus (*G*′; 13 kPa) for the TAMR/H system was found to be comparable with the agarose hydrogel of the control system. Barrangou et al. demonstrated that a 2.5% w/w agarose hydrogel possesses a *G*′ value of 6 kPa, which is comparable to the *G*′ value (13 kPa) of the hydrogel used in the TAMR/H system.^57^ The higher elastic component (*G*′) of the hydrogel used in the TAMR/H system in comparison to that of the 2.5% w/w agarose hydrogel can be attributed to the higher amount of polymer (15% w/w). All these suggest that the hydrogel used in the TAMR/H system is mechanically stable for long time periods in different solvents (Figure S4) and its elasticity or stiffness is comparable with standard diffusive media (similar to agarose hydrogels) used in common microfluidic gradient generators.

### Adhesion and Maximum Volumetric Flow Rate Testing to Estimate Bond Strength between TAMR and Hydrogel (H) Components of the TAMR/H Device

**Figure 3:**
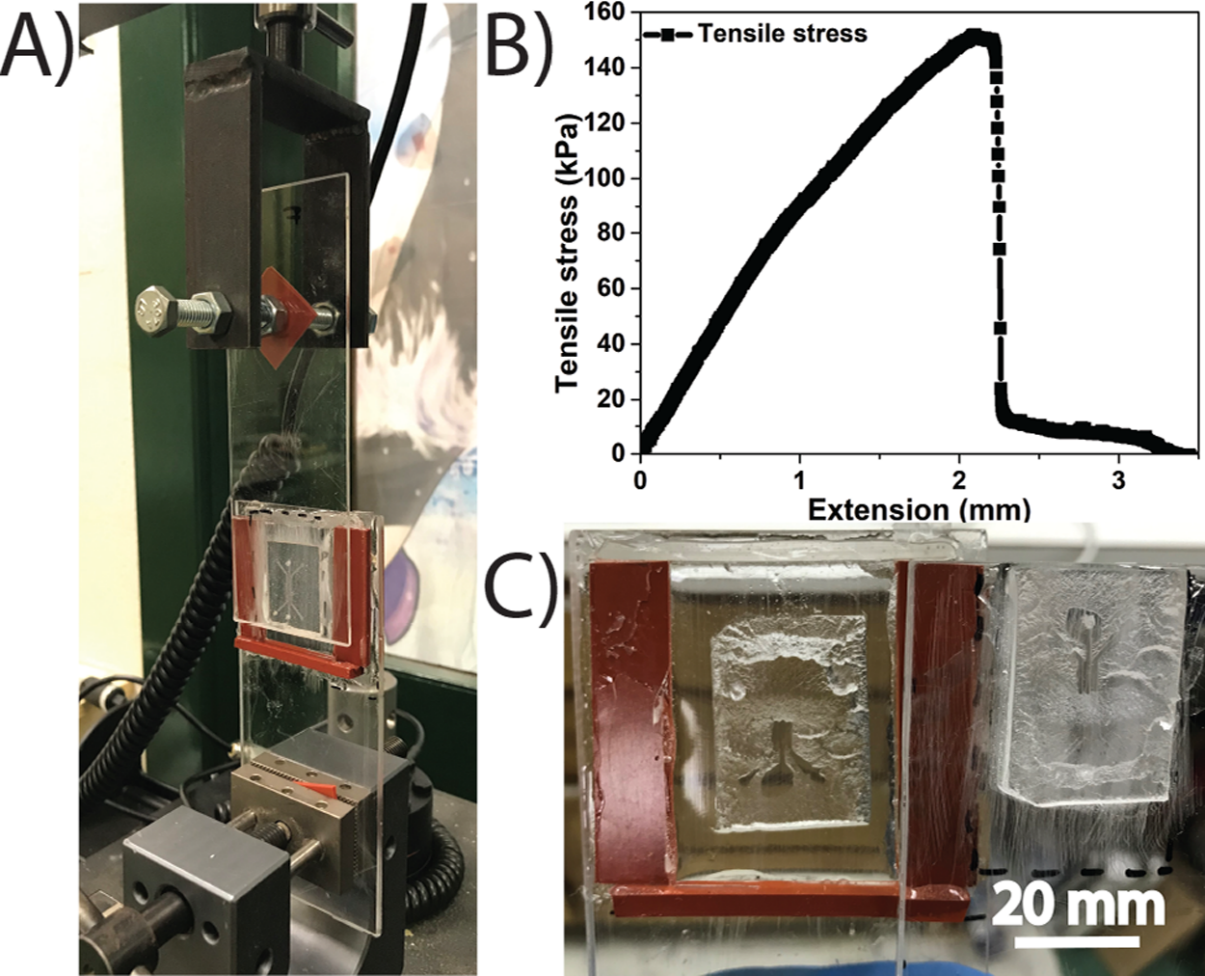
Characterization of adhesive forces between the top TAMR layer and the bottom hydrogel (H) layer. (A) Experimental setup for the lap shear test. (B) Tensile stress vs extension plot to determine minimum stress required to delaminate a TAMR/H system. (C) Example of cohesive failure (e.g., when adherent materials break due to the applied force) observed in the TAMR/H device.

An advantage of the TAMR/H device over other flow-free systems is the ability of the TAMR layer to immediately adhere to the hydrogel (H) layer. The binding strength (e.g., adhesion strength) between these two layers was evaluated using a single-lap shear test to confirm strong binding strength and evaluate the type of failure (Figures 3, S5). In this study, the TAMR and H layers were coupled with Plexiglas separately and brought into contact followed by adhesion testing using an Instron (Figures 3, S5). The strength between the two layers was approximated to be no less than 152.3 ± 6.3 kPa based on five separate trials (Figure 3B). The adhesive strength of the control PDMS/agarose system was zero (e.g., no adhesion), while standard flow devices using PDMS plasma bonded to glass have a maximum bond strength of 480 kPa as reported by Bhattacharya et al.^23,58^ Thus, while the TAMR/H device does not exhibit the strength of a PDMS/glass-bonded device, it does offer a significantly greater adhesion than PDMS/agarose or agarose/glass devices. Next, the type of failure was investigated and found to be cohesive failure. Cohesive failure occurs when adherent materials break due to the applied force, which is what was observed when the TAMR/H device was sheared with ∼152 kPa stress (Figure 3C).^23^ Moreover, the TAMR/H system was not observed to fail via adhesive failure since adhesive failure happens due to the lack of adhesion at the interface of two materials.^23^

In order to determine potential reasons for the strong adhesive force between the TAMR and hydrogel, two different formulations of TAMR (one with no excess acrylate and the other with a 50% excess acrylate group compared to thiol groups present in the TAMR system) and two different formulations of the hydrogel were synthesized (one with no excess thiol and other with 50% excess thiol groups compared to acrylate groups present in the hydrogel system). Initially, it was hypothesized that excess thiols from the hydrogel surface react with excess acrylate groups present on the TAMR surface to form a covalent bond between these two layers. However, experimentation indicated that if no excess thiol groups were present in the hydrogel (due to stoichiometric reaction) or no excess acrylate groups were present in the TAMR layer (due to stoichiometric reaction), the two layers still bound strongly since cohesive failure was observed in both systems (Figure S6B). This proves that strong adhesion between the TAMR and hydrogel did not result from the covalent bonding between excess thiol and acrylate present in the hydrogel and on the TAMR surface, respectively. Furthermore, the TAMR/H system containing 50% excess acrylate groups in the TAMR layer and no excess thiol groups in the hydrogel binds quickly (in 5 min) compared to the TAMR/H system with no excess acrylate in the TAMR layer and no excess thiol groups in the hydrogel (approximately 60 min). Therefore, it is highly likely that resultant attraction between these two layers may occur due to the hydrogen bond and dipole–dipole interaction (Figure S7). There are many hydrogen bond acceptors (e.g., nitrogen from tertiary amine covalently bonded to acrylate groups, oxygen atoms present in the carbonyl group, or the PEG group) present both in the hydrogel and on the TAMR surface and hydrogen bond donors, for example, (OH group present in the PETIA monomer) present in the TAMR layer. This can explain the reason for rapid binding between the TAMR and hydrogel in the presence of excess acrylate in the TAMR layer. Because adding excess acrylate (PETIA) in the TAMR layer means incorporating excess hydroxyl groups (e.g., hydrogen bond donors), it can ultimately increase the number of H-bond donors in the TAMR layer. Therefore, the hydrogen bond interaction between the hydrogel and TAMR containing excess acrylate occurs quickly when compared to the TAMR/H system containing no excess acrylate in the TAMR layer. These findings suggest that the reason for strong adhesion between the TAMR and hydrogel can be attributed to hydrogen bond formation and dipole–dipole interaction between these two layers (Figure S7).

**Figure 4:**
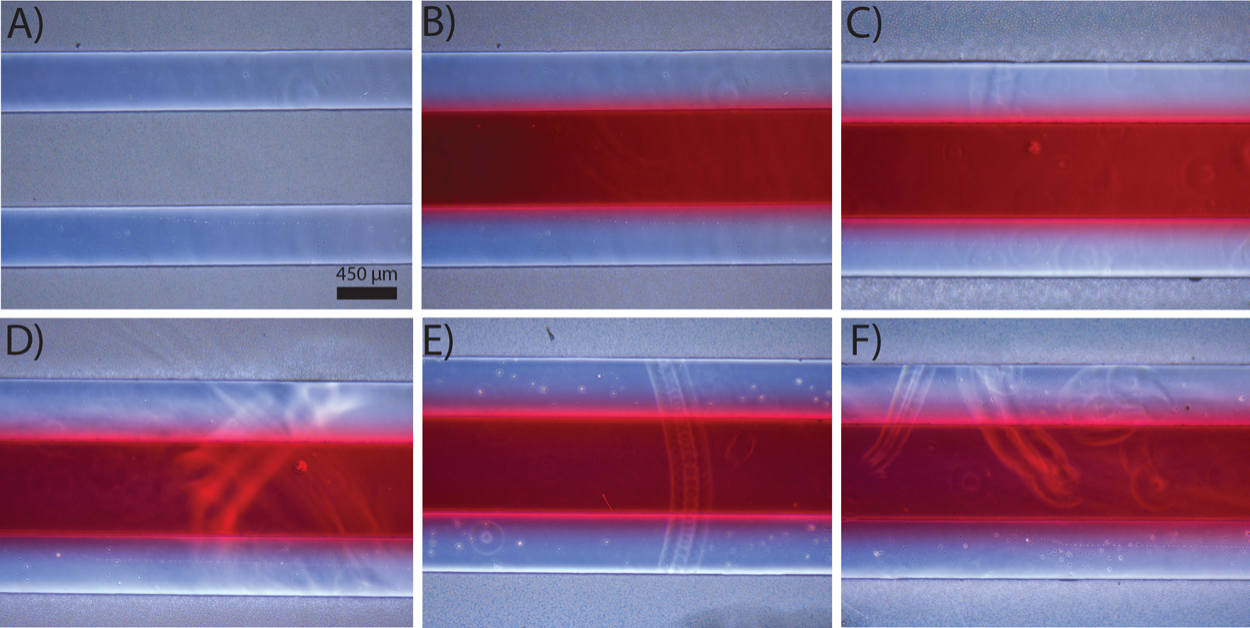
Maximum flow rate test using the TAMR/H device. (A) BF image of a TAMR/H device before flowing anything through the center channel. DI water spiked with red food dye was flown through the center channel of the assembled device at flow rates of (B) 50, (C) 100, (D) 250, (E) 500, and (F) 1000 μL/min for 5 min before acquiring the BF image using a Zeiss microscope with a 4× objective. No fluid leakage was observed at any of these flow rates. The scale bar is 450 μm for all images.

The experimental findings from the adhesion test confirmed the approximate strength of the bond between the TAMR and hydrogel layers; however, they did not provide insights into the range of fluid velocities that the device could support. A maximum volumetric flow rate test was performed using both the TAMR/H device and the control system (PDMS/agarose) to evaluate the threshold for the fluid flow rate. This control device is similar in design to previously reported flow-free gradient generators.^13,16−18^ In this test, red food dye spiked into DI water was infused into the center channel of the assembled devices in a range of volumetric flow rates to observe any fluid leakage or channel breakage due to fluid flow. No microfluidic channel breakage, or fluid leakage, was observed in the TAMR/H device up to a volumetric flow rate of 1000 μL/min (Figure 4). Moreover, the blur in the boundary of the central channel formed at increasing flow rates can be attributed to a gradient being established in the device. The differences in the observed gradients are due to the different flow rates which can be attributed to a greater amount of dye entering the hydrogel at higher flow rates. These results were encouraging as the average working volumetric flow rate of oxygen plasma-treated PDMS on glass devices is typically between 1 and 20 μL/min with values reported of 400 μL/min and above.^59,60^ Conversely, device failure and fluid leakage were observed in the middle channel in the control PDMS/agarose system at a volumetric flow rate of 135 μL/min (Figure S8). Comparing the maximum flow rate of TAMR/H with the control system, it is clear that the TAMR/H system can withstand at least 7× higher flow rate without any adhesive failure.

### Diffusion Coefficient Approximation for the Hydrogel Used in the TAMR/H System

The mass transfer rate within the hydrogel layer of the TAMR/H device was approximated by studying the diffusion of bromothymol blue (MW: 624 g/mol) in the hydrogel as a function of time. The migrating front of the bromothymol blue was observed for 500 min at different time points in the hydrogel and then fit to an error function, which approximates one-dimensional mass transfer (Figure S9) at each time point. The mass transfer values (4*Dt*) at each time point were plotted as a function of time to generate a linear relationship to approximate the diffusion coefficient (*D*) of bromothymol blue (Figure S10). The diffusion coefficient in the hydrogel was calculated to be (3.3 ± 0.2) × 10^–8^ cm^2^/s. As a comparison, the diffusion coefficient of bromothymol blue in agarose has been reported as 4.09 × 10^–6^ cm^2^/s.^50^ The lower magnitude diffusion coefficient for bromothymol blue in the TAMR/H hydrogel compared to the agarose hydrogel can be explained by the greater polymer content (15 wt %) in the TAMR/H hydrogel compared to 3 wt % agarose. This higher polymer content can increase the cross-linking density or chain entanglement that can reduce the mesh or pore size of the hydrogel.^54,55,61,62^

To investigate the tunability of the diffusion rate in the hydrogel of the TAMR/H system, different formulations of the hydrogel were synthesized using different weight percentages of the polymer (12.5 and 20 wt %). It was found that the diffusion coefficient (*D*) bromophenol blue in the hydrogel could be tuned by changing the weight percentage of the polymer present in the system varying from 1.0 × 10^–7^ to 1.4 × 10^–8^ cm^2^/s by changing weight percentage of the thiol acrylate polymer from 12.5 to 20 wt %. An observed decrease in the diffusion coefficient was observed with an increase in polymer content into the hydrogel system. This occurs because as polymer content increases in a system, the cross-linking density, or chain entanglement, increases, which reduces the mesh or pore size of that system.^54,55,61,62^ Additionally, it was found that altering the polymer content did not significantly affect the strong binding between the hydrogel and TAMR (Figure S11). An optimal weight percentage of 15.5% was used in subsequent studies due to its high chemical and mechanical stability in different solvents or any applied stress. All these suggest that both the pore size and diffusion coefficient of the hydrogel can be tuned by varying polymer content of the hydrogel. Finally, cross-sectional cryo-SEM images were collected on the 15.5 wt % hydrogel to visualize the pore size of the hydrogel used in the TAMR/H system (Figure S12). These images confirm that the hydrogel used in the TAMR/h system is porous, and pores are heterogeneous in size.

### Experimental and Computational Validation of Mass Transfer in the TAMR/H Device

**Figure 5:**
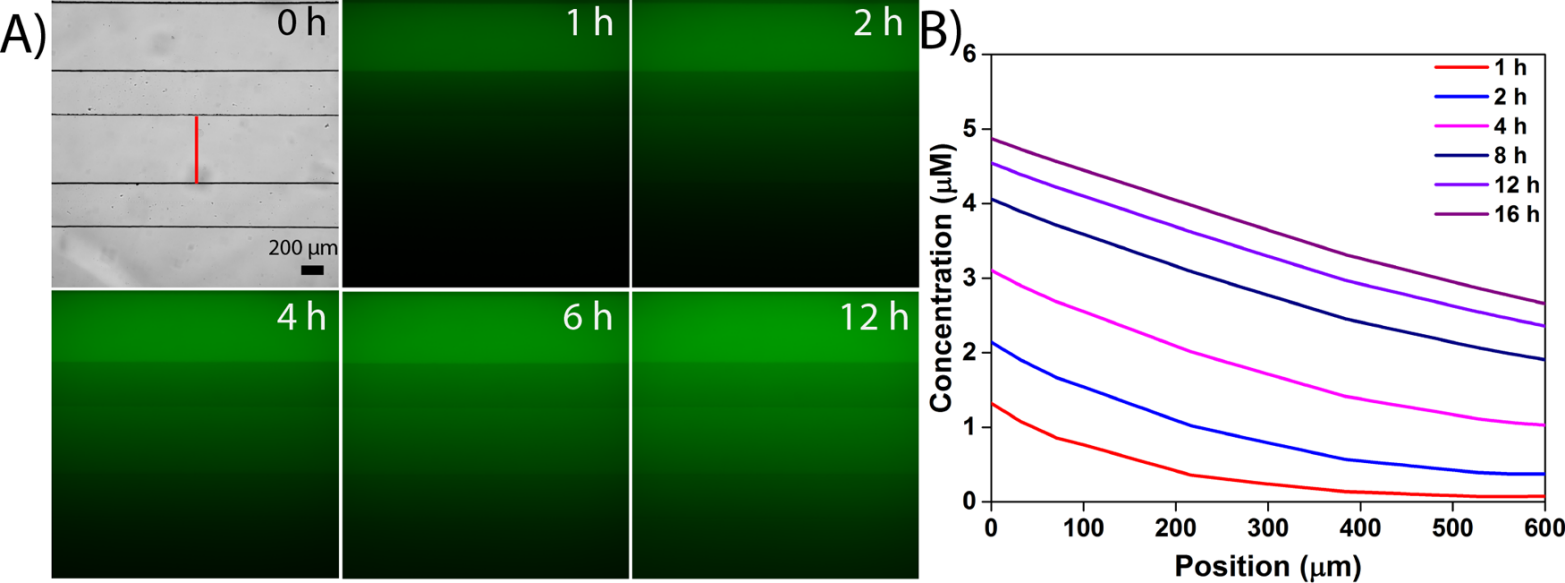
Gradient characterization in the TAMR/H microfluidic device. (A) Visualization of an orthogonal gradient of a 10 μM solution of 5,6-carboxyfluorescein (FAM) in DI water infused through the top (source) channel at a rate of 15 μL/min. A stable concentration gradient of FAM was developed after 4 h of FAM flow through the source channel. (B) Numerical simulation of the mass transfer profile (e.g., concentration gradient formed) across the center flow-free channel using COMSOL Multiphysics. TAMR/H hydrogel parameters were tuned by comparing the numerical output to fluorescent microscopy data. The value of 0 μm represents the top of the center channel closest to the source channel, while the value of 600 μm indicated the bottom of the center channel closest to the sink channel. The scale bar is 200 μm.

Fluorescence microscopy and COMSOL simulations were performed to characterize the time-dependent mass transfer of biomolecules across the TAMR/H device to confirm its ability to generate stable chemical gradients. A 10 μM solution of a model fluorescent molecule (5,6-carboxyfluorescein, FAM) was perfused in the source channel of the TAMR/H device at a rate of 15 μL/min for 16 h to visualize the chemical gradient in the center flow-free channel. FAM was chosen to model mass transfer due to its similar size (MW 376 g/mol) to several *E. coli* chemoattractants and chemorepellents.^63^ The FAM was found to easily diffuse through the TAMR/H hydrogel and into the center flow-free channel in a time-dependent manner (Figure 5A). The relative intensity and shape of the gradient were quantified by performing a line scan across the width of the center channel (Figure 5A, red line, and Figure S13). The approximate slope of this chemical gradient was found to stabilize within 4 h (Figure S13A), ultimately reaching an approximate steady state within 12 h. A uniform concentration in the center channel was avoided by continually flowing CB in the sink channel at a rate of 15 μL/min. A similar gradient characterization experiment was performed using a control microfluidic device consisting of a PDMS layer containing the three parallel fluidic channels coupled with a bottom 3% (w/w) agarose hydrogel to facilitate mass transfer (Figure S14). Experimental validation of the chemical gradient in the center flow-free channel of the PDMS/agarose yielded similar results to those found in the TAMR/H device with a gradient developing across the center channel in a time-dependent manner (Figure S14A). The gradient in the PDMS/agarose device stabilized within 2 h (Figure S13B) and reached an approximate steady state within 6 h as determined by line scan analysis. These findings are similar to those reported in the literature using a similar device.^17^ As discussed above, the difference in gradient development and stabilization time between the TAMR/H device and the PDMS/agarose device can be attributed to a greater polymer content (15% w/w) in the TAMR/H hydrogel compared to the agarose hydrogel (3% w/w).

Increasing the polymer content and cross-linking density can also alter the porosity of the hydrogel. The porosity of agarose has been reported in the literature as 0.9805; however, as the TAMR/H hydrogel is a novel material, the porosity was unknown.^64^ COMSOL simulations were performed to accomplish two goals: (1) to computational validate the observed mass transfer in the device and (2) to approximate the porosity of the hydrogel for the TAMR/H system. The COMSOL model was first developed for the PDMS/agarose device because all of the physical properties of PDMS and agarose are well known and reported in the literature. The simulation nicely matched the experimental observations of a linear gradient that developed over time (Figure S14B). A side-by-side comparison of the COMSOL values and the experimental line scans could not be performed due to the nature of the bottom layer hydrogel. Mass transfer occurs through the hydrogel perpendicular to the direction flow but also in the *z*-direction away from the flow-free channel. The bottom agarose layer was bounded by Plexiglas, which resulted in a slow accumulation of FAM in the *z*-direction compared to no accumulation at the far side of the flow-free channel due to the continual flow in the sink channel. Nevertheless, the shape of the gradient did correlate between the experimental and computational mass transfer studies which also matched published gradient characterization efforts in the PDMS/agarose device.^65^ Once the COMSOL simulation was completed, the physical parameters for porosity and diffusion coefficient were tuned to represent the TAMR/H device so that the computational results aligned with the fluorescence microscopy results (Figure 5B). The results from the simulation matched the experimental findings using a porosity of 0.2 and a diffusion coefficient of Ni^2+^ 9.5 × 10^–7^ cm^2^/s for the TAMR/H hydrogel. The diffusion coefficient (*D*) approximated from COMSOL for Ni^2+^ or FAM was similar in magnitude to the *D* value obtained for bromothymol blue (3.3 × 10^–8^ cm^2^/s) in the hydrogel of the TAMR/H system. The small difference can be attributed to differences in the experimental setup and the molecular weight difference of bromothymol blue (MW: 624 g/mol), FAM (MW: 376 g/mol), and Ni^2+^. The order of magnitude of *D* in the TAMR/H system is similar to other commonly used hydrogels including polyvinyl alcohol, polyethylene glycol (PEG), and alginate (higher weight percent; 3 wt % or more).^66−68^ The difference in porosity and diffusion coefficient can explain the difference in time for the gradient to stabilize and reach a steady state between the PDMS/agarose device and TAMR/H device.

### Study of Bacterial Chemotaxis Using Fluorescence Microscopy to Validate the Applicability of the TAMR/H Device

**Figure 6:**
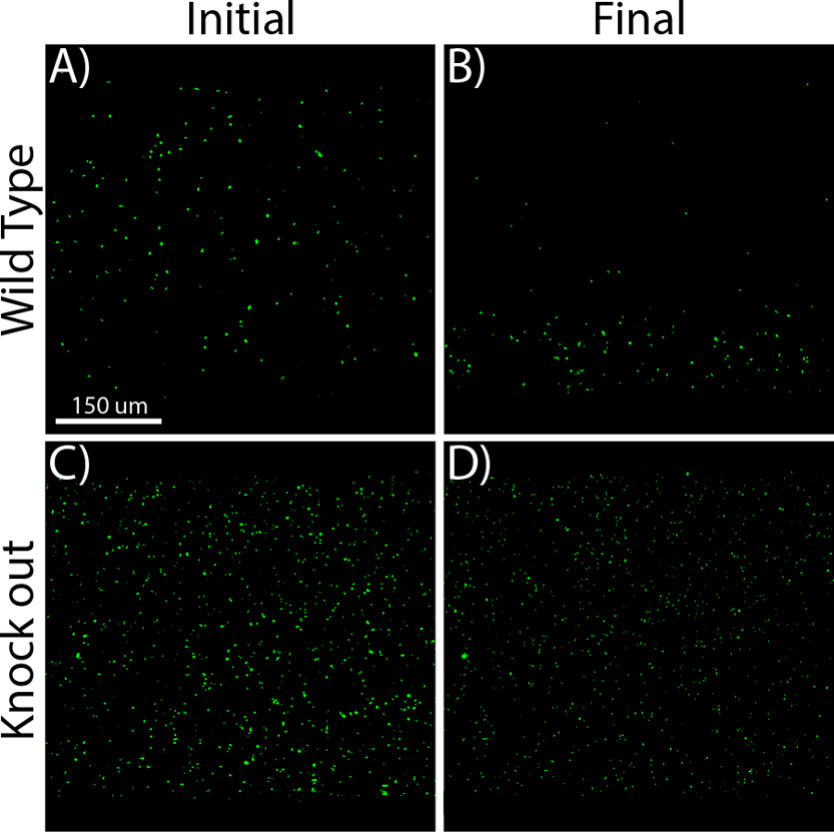
Observation of *E. coli* chemotaxis using the TAMR/H device. (A) GFP-expressing *E. coli* was seeded into the TAMR/H device after a NiSO_4_·7H_2_O gradient was allowed to develop for 3 h 40 min from the top of the channel to the bottom. NiSO_4_·7H_2_O (2 mM) was flown through the top channel at a rate of 15 μL/min. (B) Final distribution of the *E. coli* after 20 min, confirming a chemorepulsive response. (C) Nonresponsive, knock-out straining of the GFP-expressing *E. coli* was seeded into the TAMR/H device after it was primed for 3 h 40 min. (D) Final distribution of *E. coli* after 20 min, confirming no response of the KO line. Images are representative of triplicate experiments.

Bacterial chemotaxis was chosen to demonstrate a real-world application of the TAMR/H device. Two strains of GFP-expressing *E. coli* RP437 were used for the migration experiments where the knock-out strain is missing the chemoreceptor (and cannot sense the Ni^2+^ gradient), while the wild type still has the receptor to respond to the NiSO_4_·7H_2_O chemorepellent gradient.^36,40^ A NiSO_4_·7H_2_O gradient was allowed to develop across the center flow-free channel in the TAMR/H device for ∼4 h before injecting the *E. coli* into the device. Once exposed to the Ni^2+^ gradient, the cells demonstrated a chemorepulsive response toward the Ni^2+^ gradient within 20 min of exposure. As the bacterial chemorepulsive response was established, images across the length of the channel were collected to visualize the chemorepulsive response (Movie S1). The center channel was imaged at *t* = 0 min (“Initial”) and *t* = 20 min (“Final”) to quantify the migratory response of the *E. coli* (Figures 6 and S15). As expected, the wild-type strain exhibited a prominent chemotactic response away (directed migration) from the NiSO_4_·7H_2_O gradient, as evidenced by a higher density of cells at the bottom of the channel (e.g., closer to the sink channel) after 20 min. No chemotactic response was observed in the knock-out strain as evidenced by an even distribution of cells in both the initial and final images. This suggests that the observed migratory response in the device was due to chemotaxis and not random migration. Moreover, the observed *E. coli* concentration for both the knock-out (KO) and the wild type (WT) were similar across triplicate experiments (Figure S15). One difference was that the percentage of cells that express GFP was greater in the KO cells when compared to wt cells which can explain the observation of a lower number of cells between the wt and KO experiments (Figure S15). A similar experiment was performed using the control PDMS/agarose device with a similar finding between the wild-type and knock-out strains (Figures S16 and S17).

**Figure 7:**
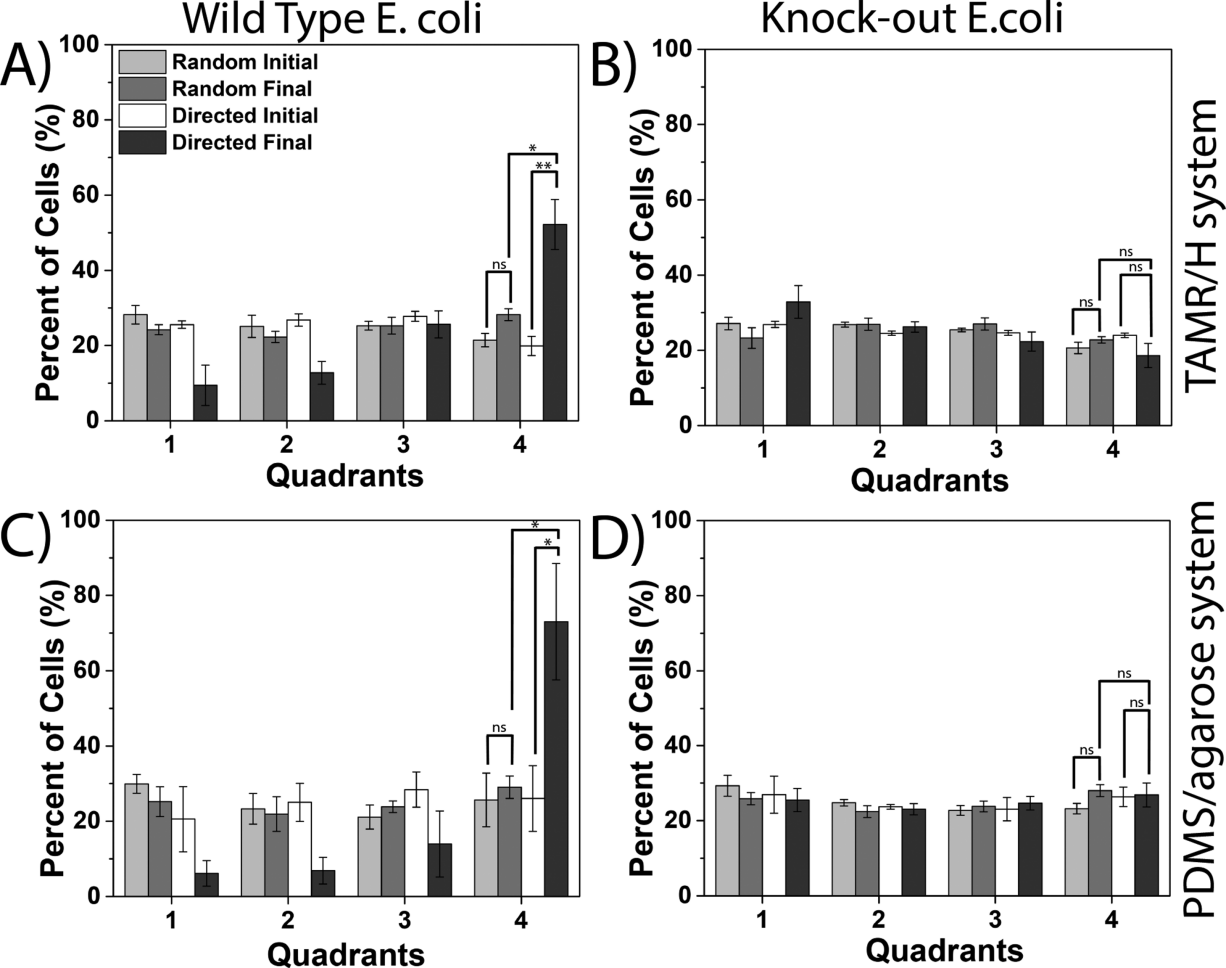
Biophysical analysis of *E. coli* migration in the microfluidic devices. The center flow-free channel was divided into four equally spaced, horizontal quadrants where quadrant 1 is closest to the chemical source (and the top channel in the device) and quadrant 4 is furthest from the chemical source. Cells were counted in each quadrant across the entire center channel and normalized against total population of cells within the device. The migratory response of wild-type (A,C) and knock-out (B,D) GFP-expressing *E. coli* was analyzed in the TAMR/H device (A,B) and the PDMS/agarose device (C,D). The *p*-value is represented in the last quadrant with comparisons between distribution. *denotes *p* < 0.05 and ** denotes *p* < 0.01, while “ns” is used when *p* > 0.05 to demonstrate nonsignificance. All experiments were performed in triplicate.

While the microscopy images are helpful to visualize the chemotactic response, a more detailed analysis is required to demonstrate the capabilities of the TAMR/H device. The center channel was binned into four equally spaced, horizontal quadrants where quadrant 1 is closest to the source (and the top channel in the device) and quadrant 4 is furthest from the chemical source. All fluorescent *E. coli* cells were counted in the four quadrants for both chemotaxis (NiSO_4_·7H_2_O in the source channel) and random migration (CB in the source channel) experiments (Figure 7). Quantitative analysis demonstrated that the directed migration of wild-type *E. coli* resulted in statistically significant (*p* < 0.01) higher populations of cells in the third and fourth quadrants, closer to the sink channel, in both the TAMR/H device (Figure 7A) and PDMS/agarose device (Figure 7C). Conversely, there was no statistically significant difference in the distribution of the wild-type *E. coli* in both devices during the random migration experiment. A parallel analysis on the knock-out mutant revealed a similar profile in both the TAMR/H (Figure 7B) and PDMS/agarose (Figure 7D) devices with a mostly uniform distribution and no statistically significant difference in cellular distribution during both chemotactic and random migration experiments (Figure 7B,D). These results confirm that the migratory behavior of the *E. coli* was in response to the NiSO_4_·7H_2_O gradient developed in the device. These findings align with prior reports investigating the chemotactic behavior of *E. coli*.^36,38^ Moreover, these studies confirm the utility of the TAMR/H device and its ability to reproduce chemotaxis studies in established flow-free microfluidic gradient generators.

## Conclusions

A TAMR and hydrogel (H) hybrid microfluidic device was successfully developed to use as an alternative approach to create flow-free chemical gradients. The TAMR/H system can be fabricated quickly due to the fast gelation time of the hydrogel, the quick curing time of TAMR, and the near immediate time it takes to develop strong adhesion between these two layers. The hydrogel was found to be mechanically stable and remain intact for a long time (18 days) and capable of achieving mass transfer of commonly used biomolecules without the need for direct flow over the fluidic channel. The performance of the TAMR/H system was compared to a control (PDMS/agarose) device with a similar geometry to study the chemorepulsive response of a motile strain of GFP-expressing *E. coli*. The population-based analysis confirmed a similar migratory response of both wild-type and mutant *E. coli* in both the microfluidic devices. All these confirmed that the TAMR/H hybrid system is a viable alternative to traditional PDMS-based microfluidic devices considering faster device preparation, cheap material, and performance. While the studies with the bacterial system were informative, the next step in this work is to assess how the TAMR/H system works with mammalian cells. An advantage of the TAMR/H system is the ability to incorporate biomolecules (e.g., an RGD peptide) to facilitate the culture of adherent cell lines without the need to pretreat the system with poly-d-lysine or other extracellular matrix proteins. Current work is underway to improve the versatility of the TAMR/H system to facilitate studies with mammalian cells.

## Supporting Information Available

Comparison of mechanical stability, diffusion rate and bonding strength for different formulations of the hydrogel layer; monomers used for synthesizing TAMR; design of a two-layer, flow-free microfluidic gradient generator; hydrogel synthesis; FTIR characterization; rheological assessment; preparation of the TAMR/H system for the adhesion test; type of failure observed in the TAMR/H system; possible bonding interaction between the TAMR and hydrogel surface; maximum flow rate test; diffusion of bromothymol blue into the hydrogel; calculation of the diffusion coefficient; characterization of adhesives forces; cryo-SEM images; gradient characterization; overlay of both brightfield and fluorescence microscopy images; and observation of E. coli chemotaxis (PDF)
E. coli (WT) chemotaxis in a TAMR/H device (AVI)

## Supplementary Material

am1c04771_si_001.pdf

am1c04771_si_002.avi
