# Supplementary material for: Development of a Flow-free Gradient Generator Using a Self-Adhesive Thiol-acrylate Microfluidic Resin/Hydrogel (TAMR/H) Hybrid System
Source: ACS Appl Mater Interfaces. 2021 Jun 3;13(23):26735–47. doi: 10.1021/acsami.1c04771 (PMC8289190; doi:10.1021/acsami.1c04771)
Supplement: Supplementary file 1 — am1c04771_si_001.pdf [file am1c04771_si_001.pdf]

Supporting information

Development of a flow-free gradient generator using a self-adhesive thiol-acrylate microfluidic resin/hydrogel (TAMR/H) hybrid system

Anowar H. Khan[1], Noah M. Smith[2], Michael P. Tullier[1], B. Seth Roberts[2], Derek Englert[3], John A. Pojman[1], and Adam T. Melvin*[2]

[1]Department of Chemistry, Louisiana State University, Baton Rouge, LA, 70803

[2]Cain Department of Chemical Engineering, Louisiana State University, Baton Rouge, LA, 70803

[3]Chemical and Materials Engineering, University of Kentucky, Paducah, KY, 42002

*Correspondence to: Adam Melvin, Cain Department of Chemical Engineering, Louisiana State University, Baton Rouge, LA, 70803. Email: melvin@lsu.edu, Phone: (225) 578-3062

Table of Contents for Supporting Information

Table S1. Comparison of mechanical stability, diffusion rate and bonding strength for different formulations of the hydrogel (H) layer

Figure S1: Monomers used for synthesizing thiol-acrylate microfluidic resin (TAMR).

Figure S2. Design of a two-layer, flow-free microfluidic gradient generator.

Schematic S1. Hydrogel synthesis

Figure S3. FTIR characterization of monomers (PEGDA, TMPETA, and ETTMP) used in the synthesis of the hydrogel of the TAMR/H system.

Figure S4. Rheological assessment of the mechanical stability of the hydrogel (H).

Figure S5. Preparation of TAMR/H system for adhesion test.

Figure S6. Type of failure observed in TAMR/H system.

Figure S7. Possible bonding interaction between TAMR and hydrogel surface.

Figure S8. Maximum flow rate test for the control PDMS/agarose device.

Figure S9. Diffusion of bromothymol blue into the hydrogel.

Figure S10. Calculation of diffusion coefficient (D) of hydrogel (H).

Figure S11. Characterization of adhesives forces between the TAMR and the hydrogel (H) layers.

Figure S12. Cryo-SEM images of cross section of the 15.5 wt. % hydrogel (H).

Figure S13. Gradient characterization in the TAMR/H and PDMS/agarose microfluidic device using line scan.

Figure S14. Gradient characterization in the PDMS/agarose microfluidic device.

Figure S15. Overlay of both brightfield and fluorescence microscopy images of *E. coli* chemotaxis using the TAMR/H device

Figure S16. Observation of *E. coli* chemotaxis using the PDMS/agarose device.

Figure S17. Overlay of both brightfield and fluorescence microscopy images of *E. coli* chemotaxis using the PDMS/agarose device

Movie S1. *E. coli* (WT) chemotaxis in a TAMR/H device.

SUPPLEMENTAL METHODS

Wafer fabrication

The TAMR/H device geometry as designed in AutoCAD (Autodesk, USA) to generate a transparency mask (CAD/Art) of the three fluidic channels. A silicon master wafer was fabricated by depositing SU-8 2050 (Kayaku Advanced Materials), a negative photoresist polymer, onto a 3” silicon wafer (University Wafer) using a spin coater (WS-650MZ-23NPP, Laurell, USA) to generate a final height of 150 μm by utilizing a two-step process. First, a 75 μm-thick layer of SU-8 2050 was deposited onto the wafer, followed by baking at 65 °C and 95 °C for 10 min and 20 min, respectively. Next, the second layer of SU-8

2050 (also 75 μm thick) was deposited on the wafer, followed by baking at the same conditions. After the wafer was cooled to room temperature, the transparency mask was placed on top of the wafer, followed by exposure to UV light (1.4 mW/cm^2) for 128 s in a custom-built UV exposure set-up using a B100-AP lamp (VWR). The wafer was baked at 65 °C for 15 min and at 95 °C for 2 h after UV exposure. After cooling at 25 °C for 30 min, SU-8 developer (Kayaku Advanced Materials) was used to remove all uncrosslinked SU-8. The wafer was hard-baked at 150 °C for 1 h to increase durability and then treated with a silane (tridecafluoro-1,1,2,2-tetrahydrooctyl trichlorosilane, Sigma-Aldrich) in a vacuum to deposit a thin layer on the surface of the wafer, which facilitates the detachment of the polymeric replicas. The same wafer was used to generate both TAMR and PDMS replicas of the fluidic channels.

Synthesis of thiol-acrylate microfluidic resin (TAMR)

15.3% activated pentaerythritol tri and tetra (1:1) acrylate (PETIA, Mn 325) or amine conjugated PETIA was synthesized by mixing 100 g PETIA with 12 g diethylamine (DEA) in a 250 mL polypropylene (PP) container and the mixture was stirred on a magnetic stir

plate for 3 h at room temperature for complete conversion. After activating, 15 g of activated PETIA was quickly (~10-15 s) mixed in a weighing boat with 12 g trimethylolpropane tris(3-mercaptopropionate) (TMPTMP, Mw 398.5) and transferred into a 50 mL Teflon tube to centrifuge at 2500xg for 30 seconds for removing any bubbles trapped inside the reaction mixture. After centrifugation, the bubble-free TAMR mixture was immediately poured over the silicon wafer and allowed it to cure at room temperature for five to six minutes before removing it from the wafer.

Fabrication and assembly of the PDMS/agarose microfluidic device

The control microfluidic device (Figure S2) contained two layers: a bottom layer of 3% (w/v) agarose (Invitrogen) to facilitate chemical diffusion and a top PDMS (Sylgard 184, Dow) layer. PDMS replicas were generated by mixing 25 g of the base and 2.5 g of the curing agent in a 10:1 ratio followed by degassing in a vacuum chamber to create a bubble-free mixture. The PDMS was poured on the silicon master wafer and was allowed to cure for at least 12 h at 65°C. Cured replicas were removed from the wafer and individual devices were cut out with an X-Acto knife, followed by punching the inlet and

outlet ports using a blunted 18-gauge needle. The PDMS and agarose components were fixed in place using two rectangular pieces of Plexiglas (McMaster-Carr) held together by four screws at the four corners of the device (Figure S2B). The thickness of the agarose layer was ~3 mm and the thickness of the PDMS layer was ~3 mm, as measure by a caliper to ensure that the two layers remained fluid tight.

FTIR characterization of hydrogel (H)

The method performed here are similar to previous work by Khan et al.[1] Water from the hydrogel samples was replaced with acetone by soaking it serially in 25, 50, 75 and 100% acetone for 30 minutes each followed by drying the samples at room temperature (25°C) overnight in negative 1 atm pressure. FTIR spectra of monomers (PEGDA, TMPETA and ETTMP) and dried hydrogel were collected using a Bruker Tensor 27 FTIR spectrophotometer equipped with a Pike Miracle single bounce diamond attenuated total reflectance (ATR) cell.

Evaluation of the mechanical properties of the swelled hydrogel used in TAMR/H system

For long-term experiments, the uncovered hydrogel surface of TAMR/H system were covered with deionized (DI) water to prevent any water evaporation from the hydrogel matrix. Therefore, it was necessary to test whether keeping the hydrogel under DI water or chemotactic buffer (CB) had any effect on the mechanical properties of the hydrogel. 5 g of freshly prepared hydrogel was submerged under 100 mL of CB or DI water and kept at 37℃ for 12 days before running rheological measurement for the storage modulus (G’). It was found that the storage modulus (G’) or elastic modulus of the hydrogel remains approximately the same (Figure S4) compared to the G’ of freshly prepared hydrogel (Figure 2B, C). This indicates that the crosslinking density of the hydrogel does not decrease significantly even if the hydrogel sample was kept under CB or DI water for several days (12 days) since crosslinking density is proportional to G’.[1-2] Therefore, keeping uncovered hydrogel surface of TAMR/H system under CB or DI water should not affect mechanical properties of the hydrogel at least for 12 days.

Cryo-SEM imaging to visualize the morphology of the hydrogel

Cryogenic scanning electron microscopy (Cryo-SEM) was performed on Hitachi S-4800 SEM equipped with Gatan Alto 2500 cryogenic SEM system. In a typical procedure, a hydrogel (H) sample was loaded into a cylinder device and formed a drop on the cylinder top. The cylinder was attached onto a stub. The stub was plunged into slushed liquid nitrogen and slushed the liquid nitrogen under vacuum again. The stub was then transferred under vacuum to the cold stage (at −130 °C) of the pre-chamber connecting to the SEM chamber. The frozen sample was surface fractured and sublimated at −95 °C for 15 min to reveal the cross-sectional surface. The temperature was then brought down to −130 °C and the sample was sputter coated with platinum-palladium before being transferred under vacuum into the SEM chamber, which was kept at −130 °C for imaging. The fracture surface was observed at accelerating voltage 3 kV.

Table S1. Comparison of mechanical stability, diffusion rate and bonding strength for different formulations of the hydrogel (H) layer

Different wt.% hydrogel (H)	Diffusion Coefficient (cm^2/s)	Mechanical stability of the hydrogel in dH_2O	Adhesion strength of TAMR and H is no less than (kPa)	Type of failure of the TAMR/H system
12.5	1.0×10^{-7}	Stable	76	Cohesive failure
15	3.3×10^{-8}	Stable	152	Cohesive failure
20	1.4×10^{-8}	Stable	120	Cohesive failure

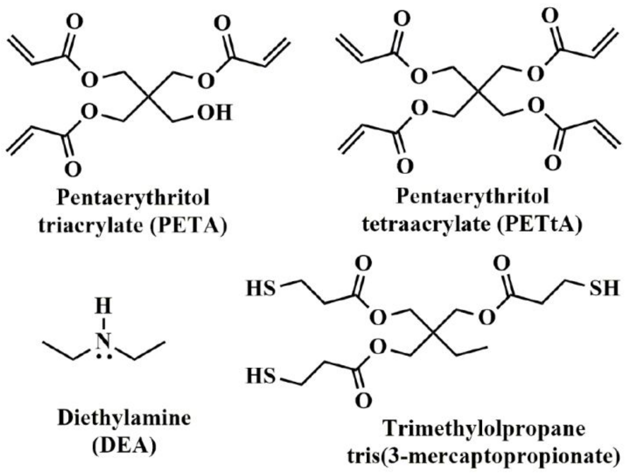

Figure S1. Monomers used for synthesizing thiol-acrylate microfluidic resin (TAMR).

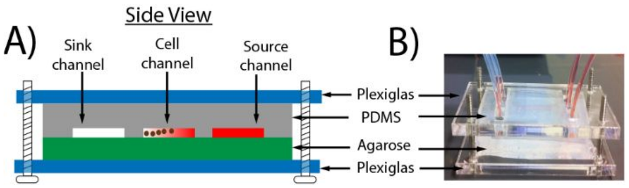

Figure S2. Design of a two-layer, flow-free microfluidic gradient generator. A) Schematic illustration of PDMS/agarose device showing its different component. B) An actual PDMS/agarose device.

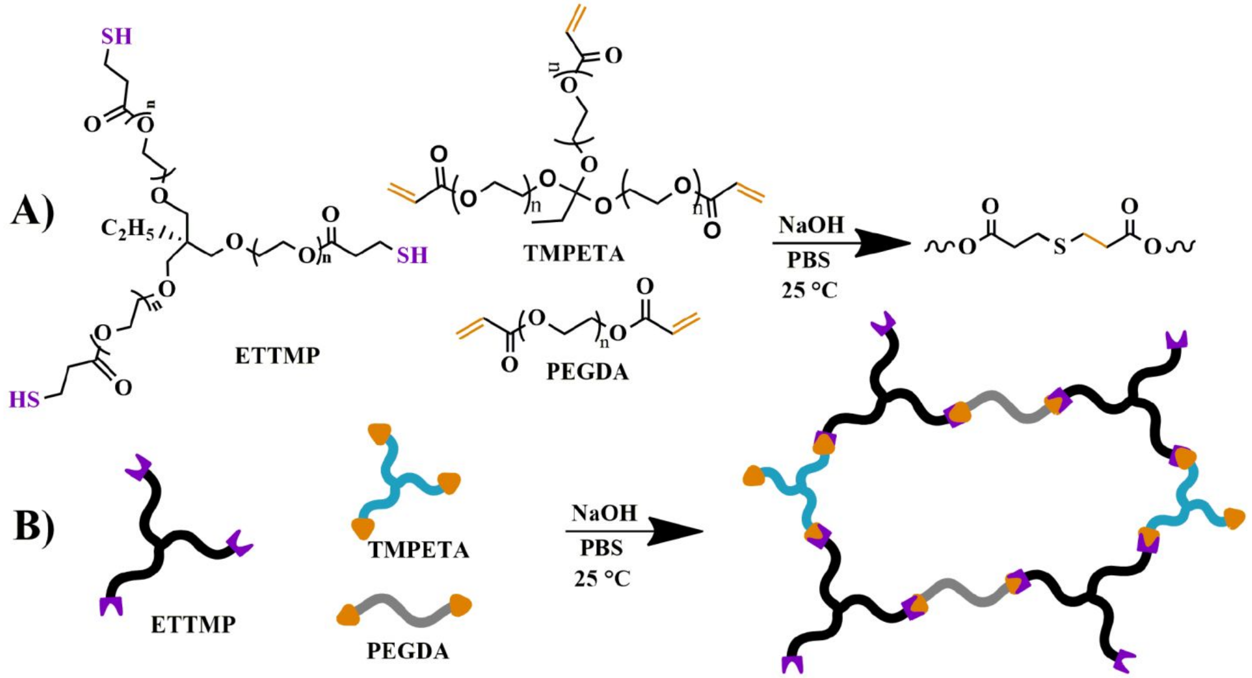

Schematic S1. Hydrogel synthesis. A) Synthesis scheme of hydrogels generated by a base-catalyzed Michael addition at room temperature. B) Schematic representation of thiol (ETTMP) and crosslinker acrylate (PEGDA, TMPETA) monomers to generate the final crosslinked polymer chains in the in the TAMR/H hydrogel.

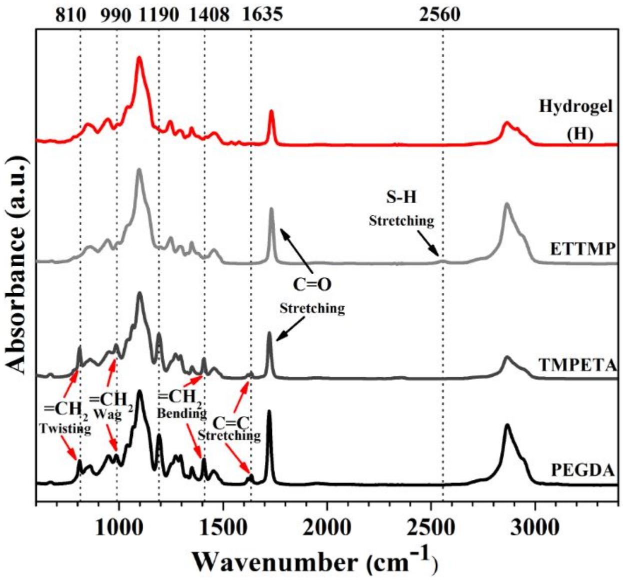

Figure S3. FTIR characterization of monomers (PEGDA, TMPETA, and ETTMP) used in the synthesis of hydrogel of the TAMR/H system. IR bands located at (indicated by red and black arrows) 2560, 1635, 1408, 990, 810 cm^{-1} can be indexed to S-H stretching (thiol), C=C stretching (acrylate), $=CH_2$ bending, $=CH_2$ wagging, and $=CH_2$ twisting respectively were disappeared from the hydrogel (H) spectrum. This confirmed that the hydrogel was successfully synthesized via Michael addition.

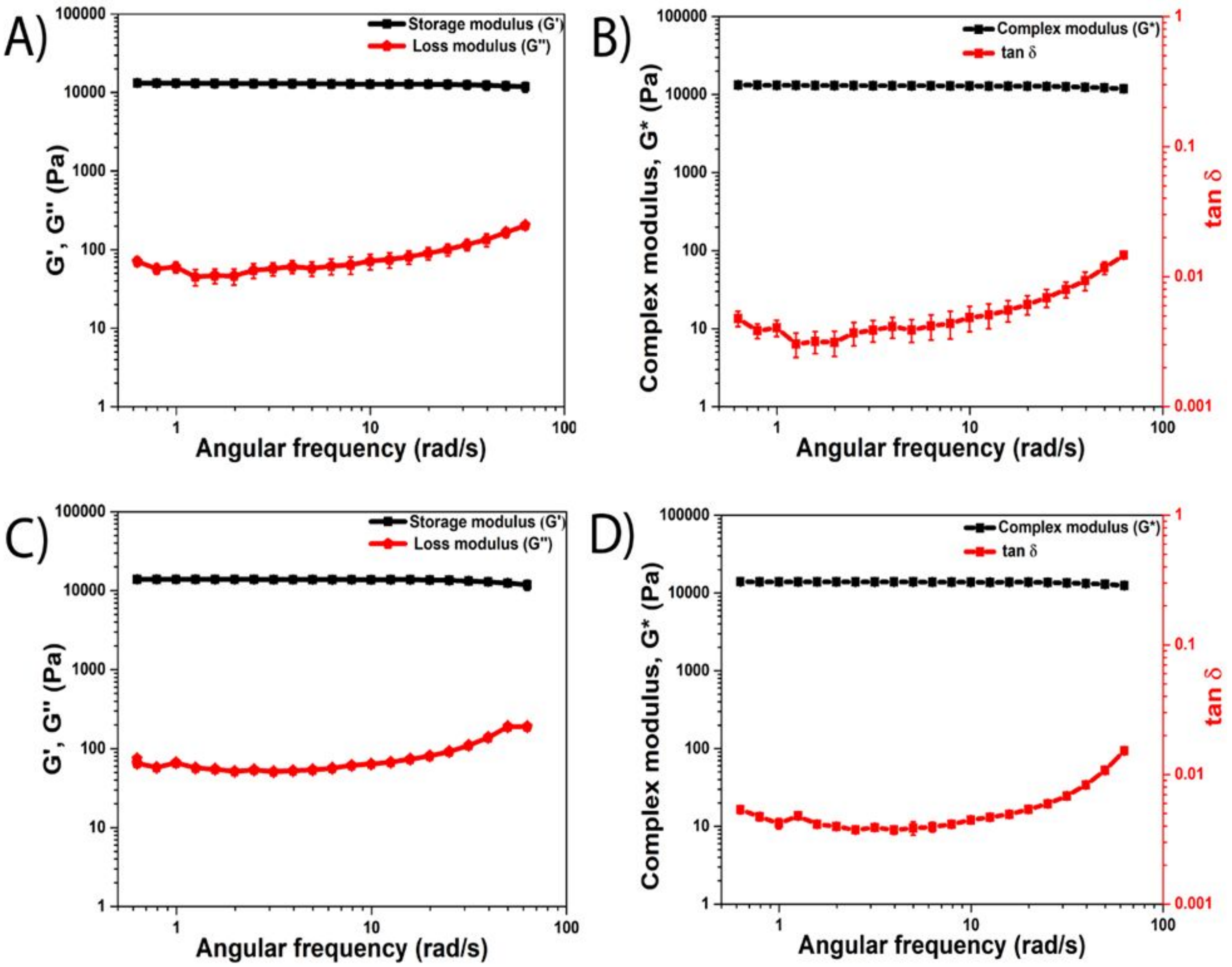

Figure S4. Rheological assessment of the mechanical stability of the hydrogel (H). The hydrogel was maintained in either chemotactic buffer (CB, pH = 7.41) or deionized (DI) water (pH = 8.15) at 37 °C for 12 days. Bulk rheology of covalently crosslinked thiol-acrylate hydrogel (H) was taken during frequency sweeps (0.682 to 62.8 radians/second) at 25°C. A comparison between the storage modulus (G'; elastic component) and the loss modulus (G''; viscous component) during the frequency sweep when the sample was kept

under CB (A) and DI water (C) for 12 days. Comparison between the complex shear modulus G* (provides insight on hydrogel stiffness) and tan δ (tan δ ~ 0: purely elastic material and tan δ ~ 1: viscous liquid) during the frequency sweep when the sample was kept under CB (B) and DI water (D) for 12 days.

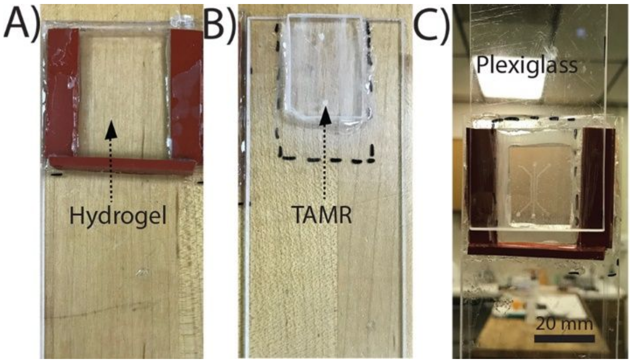

Figure S5. Adhesion testing of the TAMR/H system to determine the approximate strength of the bond. A) The hydrogel was synthesized over a Plexiglas surface which easily adhered to the surface. B) A 22x32 mm TAMR layer was glued to the Plexiglas surface using five-minute epoxy. C) Both layers were brought in contact for 5 minutes which resulted strong adhesion and ready to be tested.

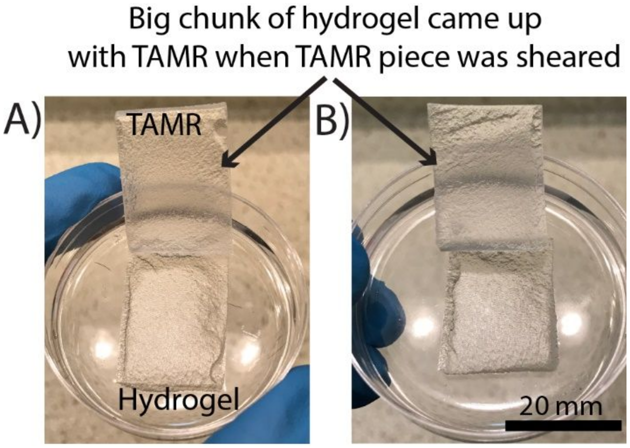

Figure S6. Cohesive failure (e.g., when adherent materials break due to the applied force) was observed in TAMR/H system made with A) 50% excess acrylate group present in TAMR with stoichiometric hydrogel or B) Stoichiometric TAMR and stoichiometric hydrogel.

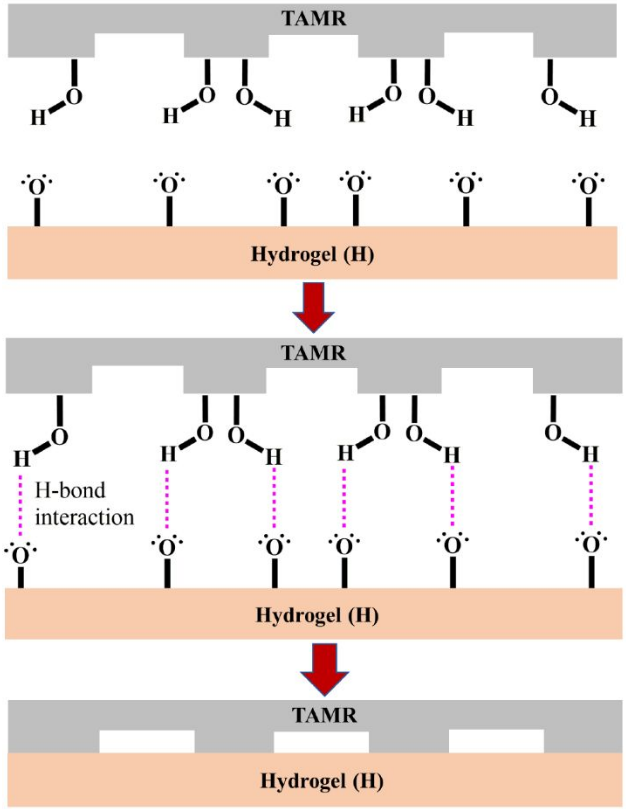

Figure S7. Possible bonding interaction between TAMR and hydrogel surface. Hydrogen bond donor (OH group present in PETIA monomer) present in TAMR layer interacts with hydrogen bond acceptor (Oxygen atom present in carbonyl group or PEG group, nitrogen from tertiary amine covalently bonded to acrylate groups) present in hydrogel layer to form strong H-bond interaction which may result in strong adhesion between TAMR and H.

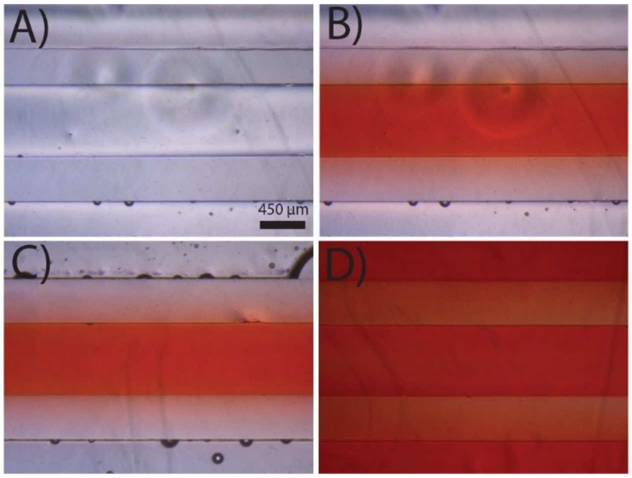

Figure S8. Maximum flow rate test for the control PDMS/agarose device. A) Brightfield image of an PDMS/agarose device before flowing anything through center channel. DI water spiked with red food dye was flown through the center channel of assembled device at a flow rate of (B) 25 µL/min, (C) 80 µL/min, and (D) 135 µL/min for 5 minutes before acquiring the brightfield image using Zeiss microscope at a 4X objective. No fluid leakage was observed at (B) 25 µL/min and (C) 80 µL/min of flow rate. A complete failure or fluid leakage from middle channel was seen at a flow rate of 135 µL/min or above. Scale bar is 450 µm for all images.

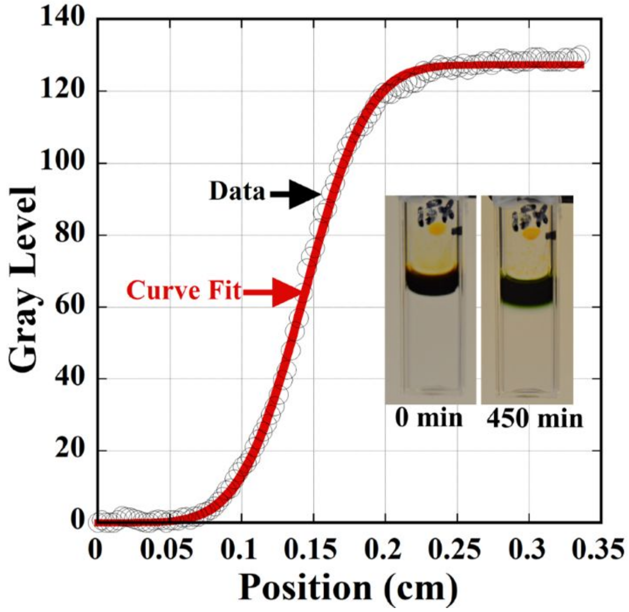

Figure S9. Diffusion of bromothymol blue into the hydrogel. The mass transfer of the dye is modeling by an error function that was numerically fit to the data (Data is for 30 min time point) using KaleidaGraph to calculate the diffusion coefficient (D). (Inset) Images of the migrating front of the bromothymol blue dye in hydrogel at 0 and 450 min.

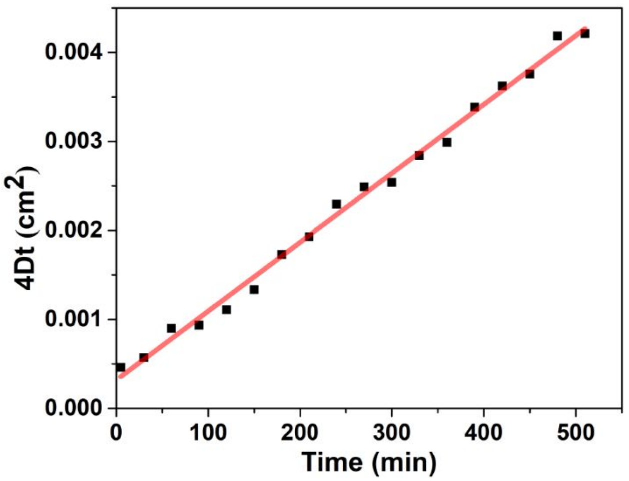

Figure S10. Calculation of diffusion coefficient (D) of hydrogel (H). At each time point, the value of 4Dt was calculated from a mathematical model of one-dimensional mass transfer fit to an error function and from the slope of each line D was measured for individual formulations. From the analysis, diffusion coefficient (D) of bromothymol blue into the hydrogel (H) was found to be (3.3 ± 0.2) × 10^{-8} cm^2/s.

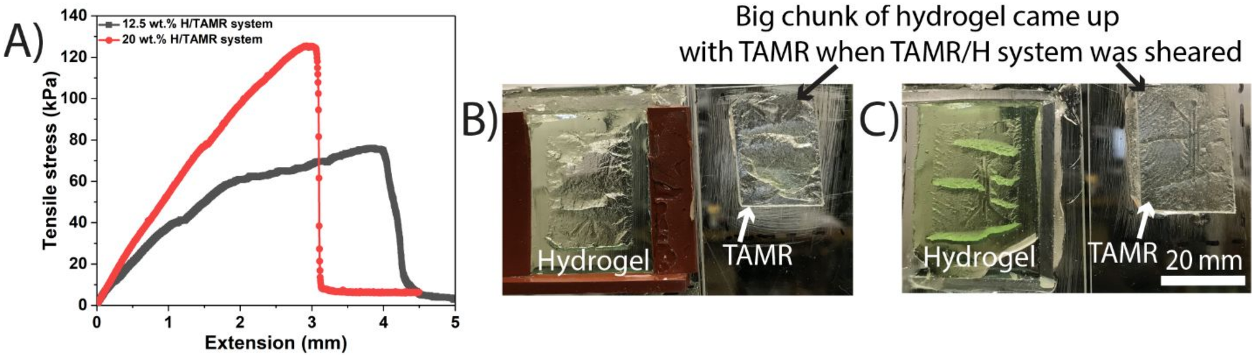

Figure S11. Characterization of adhesives forces between the TAMR and the hydrogel (H) layers. A) Tensile stress vs extension plot to determine the minimum stress required to delaminate the TAMR/H system. Example of cohesive failure (e.g., when adherent materials break due to the applied force) observed in the TAMR/H system made with 12.5 wt.% (B) and 20 wt.% (C) hydrogels.

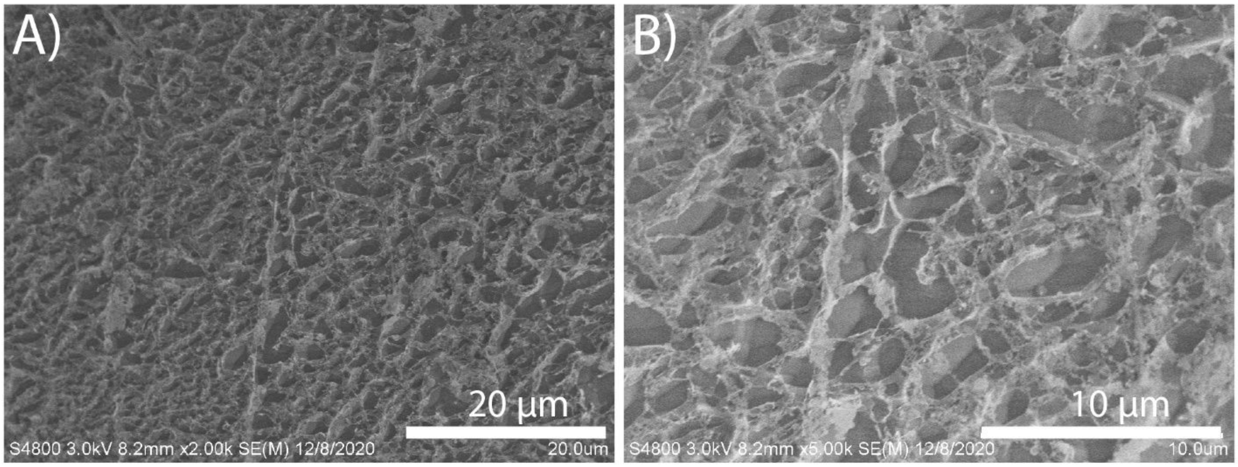

Figure S12. Cryo-SEM images of cross section of the 15 wt. % hydrogel at low magnification (A) and high magnification (B). Morphology of the hydrogel proves the presence of pores with heterogeneous sizes.

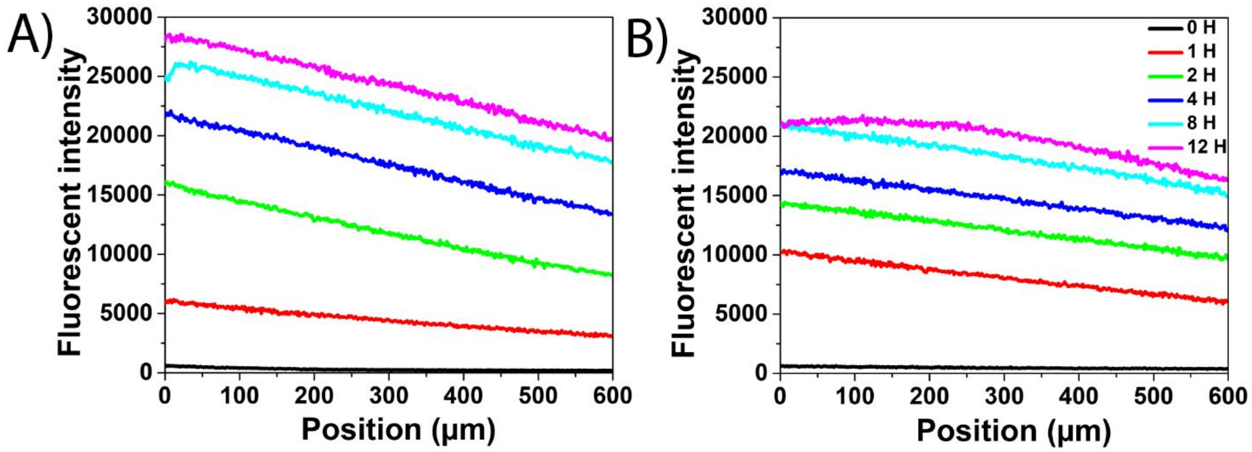

Figure S13. Gradient characterization in the TAMR/H and PDMS/agarose microfluidic device using line scan. Changes in fluorescent intensity across the center flow-free channel of the (A) TAMR/H and (B) PDMS/agarose device were obtained by performing a line scan on the fluorescence microscopy images acquired at different time intervals. The value of 0 μm represents the top of the center channel closest to the source channel while the value of 600 μm indicates the bottom of the center channel closest to the sink channel.

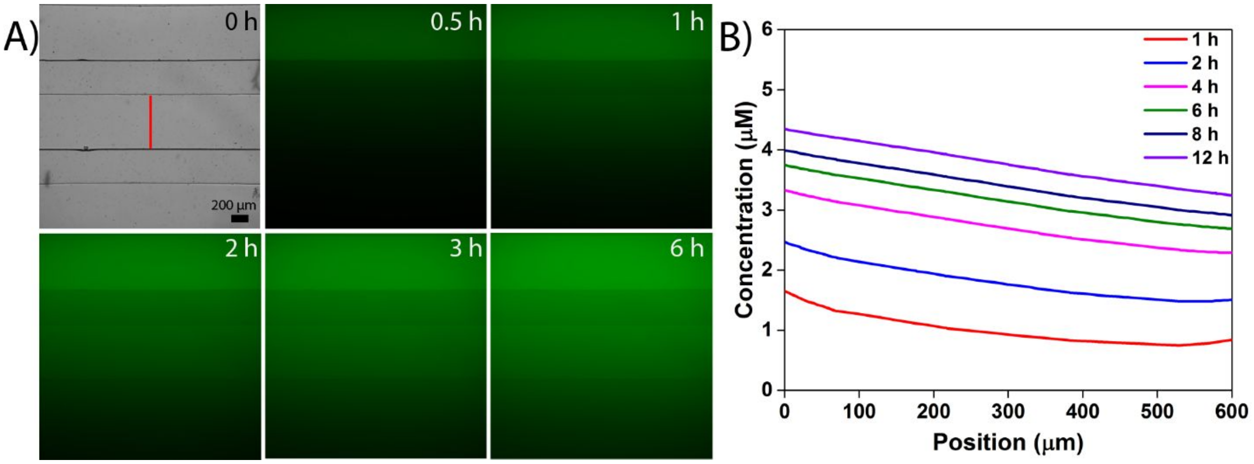

Figure S14. Gradient characterization in the PDMS/agarose microfluidic device. (A) Visualization of an orthogonal gradient of a 10 µM solution of 5,6-carboxyfluorescien (FAM) infused through the top channel at a rate of 15 µL/min. A stable concentration gradient of FAM was developed after 2 hours of FAM flow through source channel. (B) Numerical simulation of the mass transfer profiler (e.g., concentration gradient formed) across the center flow-free channel using COMSOL Multiphysics. Hydrogel parameters (e.g., diffusion coefficient and porosity) were manipulated by comparing numerical output to fluorescent microscopy data. The value of 0 µm represents the top of the center channel closest to the source channel while the value of 600 µm indicated the bottom of the center channel closest to the sink channel. Scale bar is 200 µm.

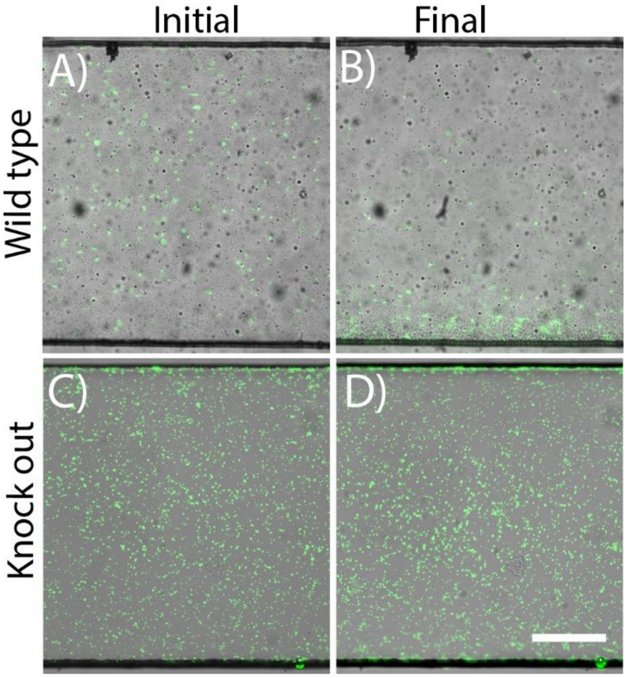

Figure S15. Overlay of both brightfield and fluorescence microscopy images of *E. coli* chemotaxis using the TAMR/H device. (A) Green fluorescent protein (GFP)-expressing

E. coli were seeded into the TAMR/H device after a $NiSO_4•7H_2O$ gradient was allowed to develop for 3 hours 40 min from the top of the channel to the bottom. 2 mM $NiSO_4•7H_2O$ was flown through the top channel at a rate of 15 µL/min. (B) Final distribution of the *E. coli* after 20 min confirming a chemorepulsive response. (C) A non-responsive, knock-out (KO) straining of the GFP-expressing *E. coli* was seeded into the TAMR/H device after it was primed for 3 hours 40 min. (D) Final distribution of *E. coli* after 25 min confirming no response of the KO line. Images are representative of triplicate experiments.

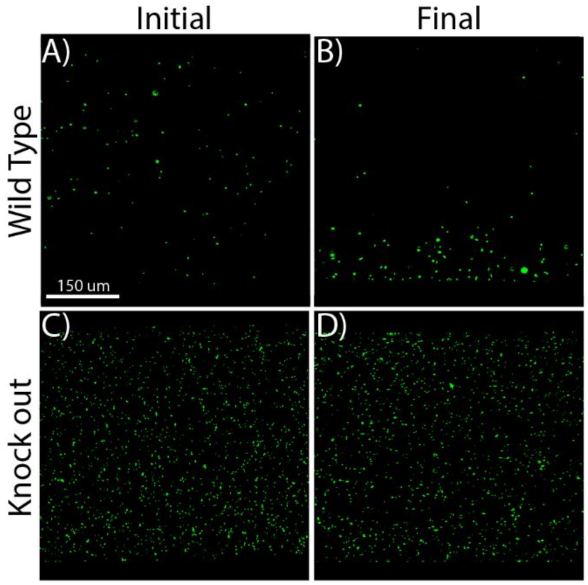

Figure S16. Observation of *E. coli* chemotaxis using the PDMS/agarose device. (A) Green fluorescent protein (GFP)-expressing *E. coli* were seeded into the PDMS/agarose device after a $NiSO_4$•$7H_2O$ gradient was allowed to develop for 1 hour 50 min from the top of the channel to the bottom. 2 mM $NiSO_4$•$7H_2O$ was flown through the top channel at a rate of 15 µL/min. (B) Final distribution of the *E. coli* after 20 min confirming a chemorepulsive response. (C) A non-responsive, knock-out straining of the GFP-expressing *E. coli* was seeded into the PDMS/agarose device after it was primed for 1 hr 50 min. (D) Final distribution of *E. coli* after 25 min confirming no response of the KO line. Images are representative of triplicate experiments.

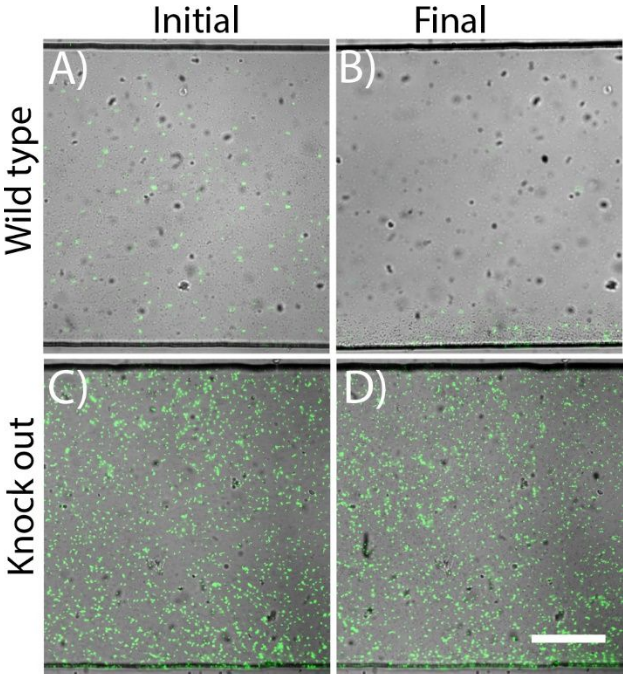

Figure S17. Overlay of both brightfield and fluorescence microscopy images of *E. coli* chemotaxis using the PDMS/agarose device. (A) Green fluorescent protein (GFP)-expressing *E. coli* were seeded into the PDMS/agarose device after a $NiSO_4$•$7H_2O$

gradient was allowed to develop for 1 hour 50 min from the top of the channel to the bottom. 2 mM $NiSO_4 \cdot 7H_2O$ was flown through the top channel at a rate of 15 µL/min. (B) Final distribution of the *E. coli* after 20 min confirming a chemorepulsive response. (C) A non-responsive, knock-out (KO) straining of the GFP-expressing *E. coli* was seeded into the PDMS/agarose device after it was primed for 1 hr 50 min. (D) Final distribution of *E. coli* after 25 min confirming no response of the KO line. Images are representative of triplicate experiments. Scale bar is 150 µm.

Movie S1: Observation of *E. coli* (WT) chemotaxis in a TAMR/H device. Green fluorescent protein (GFP)-expressing *E. coli* were seeded into the TAMR/H device after Ni^{2+} gradient was allowed to develop for 3 hours 40 min from the top of the channel (source) to the bottom channel (sink).

References

(1) Khan, A. H.; Cook, J. K.; Wortmann III, W. J.; Kersker, N. D.; Rao, A.; Pojman, J. A.; Melvin, A. T. Synthesis and Characterization of Thiol-Acrylate Hydrogels Using a Base-Catalyzed Michael Addition for 3d Cell Culture Applications. *J. Biomed. Mater. Res. Part B Appl. Biomater.* **2020,** *108*, 2294-2307.

(2) Zustiak, S. P.; Leach, J. B. Hydrolytically Degradable Poly(Ethylene Glycol) Hydrogel Scaffolds with Tunable Degradation and Mechanical Properties. *Biomacromolecules* **2010,** *11*, 1348-1357.
